# Reversal of Endothelial Extracellular Vesicle-Induced Smooth Muscle Phenotype Transition by Hypercholesterolemia Stimulation: Role of NLRP3 Inflammasome Activation

**DOI:** 10.3389/fcell.2020.597423

**Published:** 2020-12-21

**Authors:** Xinxu Yuan, Owais M. Bhat, Arun Samidurai, Anindita Das, Yang Zhang, Pin-Lan Li

**Affiliations:** ^1^Department of Pharmacology and Toxicology, School of Medicine, Virginia Commonwealth University, Richmond, VA, United States; ^2^Pauley Heart Center, Department of Internal Medicine, Virginia Commonwealth University, Richmond, VA, United States; ^3^Department of Pharmacological and Pharmaceutical Sciences, College of Pharmacy, University of Houston, Houston, TX, United States

**Keywords:** extracellular vesicles, endothelial cells, acid ceramidase, ceramide, lysosome

## Abstract

Recent studies reported that vascular endothelial cells (ECs) secrete NLR family pyrin domain-containing 3 (NLRP3) inflammasome products such as interleukin-1β (IL-1β) via extracellular vesicles (EVs) under various pathological conditions. EVs represent one of the critical mechanisms mediating the cell-to-cell communication between ECs and vascular smooth muscle cells (VSMCs). However, whether or not the inflammasome-dependent EVs directly participate in the regulation of VSMC function remains unknown. In the present study, we found that in cultured carotid ECs, atherogenic stimulation by oxysterol 7-ketocholesterol (7-Ket) induced NLRP3 inflammasome formation and activation, reduced lysosome-multivesicular bodies (MVBs) fusion, and increased secretion of EVs that contain inflammasome product IL-1β. These EC-derived IL-1β-containing EVs promoted synthetic phenotype transition of co-cultured VSMCs, whereas EVs from unstimulated ECs have the opposite effects. Moreover, acid ceramidase *(AC)* deficiency or lysosome inhibition further exaggerated the 7-Ket-induced release of IL-1β-containing EVs in ECs. Using a Western diet (WD)-induced hypercholesterolemia mouse model, we found that endothelial-specific *AC* gene knockout mice (Asah1^fl/fl^/EC^Cre^) exhibited augmented WD-induced EV secretion with IL-1β and more significantly decreased the interaction of MVBs with lysosomes in the carotid arterial wall compared to their wild-type littermates (WT/WT). The endothelial *AC* deficiency in Asah1^fl/fl^/EC^Cre^ mice also resulted in enhanced VSMC phenotype transition and accelerated neointima formation. Together, these results suggest that NLRP3 inflammasome-dependent IL-1β production during hypercholesterolemia promotes VSMC phenotype transition to synthetic status *via* EV machinery, which is controlled by lysosomal *AC* activity. Our findings provide novel mechanistic insights into understanding the pathogenic role of endothelial NLRP3 inflammasome in vascular injury through EV-mediated EC-to-VSMC regulation.

## Introduction

Vascular endothelial cells (ECs) and vascular smooth muscle cells (VSMCs) are two major cell types in the blood vessel walls, and the interplay between two cell types is critical in maintaining vascular homeostasis under physiological and pathological conditions (Bergers and Song, 2005; Li et al., 2018; Lyle and Taylor, 2019). Dysfunction of ECs is associated with the VSMC phenotypic transition toward a synthetic phenotype with enhanced proliferation and migration, which promotes the initiation and development of atherosclerotic plaques (Peiro et al., 1995; Chistiakov et al., 2015b; Kim et al., 2019). ECs and VSMCs have evolved various modes of interaction to regulate vascular function and maintain homeostasis. Theses interactions are through direct contact (Liebner et al., 2006; Pitulescu and Adams, 2014) or indirectly through releasing various mediators such as endothelial NO synthase-derived nitric oxide (eNOS-derived NO) (Yu et al., 2012), extracellular matrix (ECM) (Wagenseil and Mecham, 2009; Lutter et al., 2012), extracellular vesicles (EVs) (Hafiane and Daskalopoulou, 2018), and other factors affecting VSMCs phenotype transition (Qi et al., 2011; Korn et al., 2014). Among them, EVs describe lipid membrane-enclosed vesicles released into the extracellular space by most cell types, which plays an essential role in cell-to-cell communication. EVs include three distinct particles such as exosomes, microparticles or microvesicles, and apoptotic bodies (Noble et al., 2020; Swatler et al., 2020). Recent studies reported that VSMC proliferation and migration are regulated by EVs derived from ECs (Ryu et al., 2019) as well as from other sources including plasma (Otani et al., 2020), fibroblasts (Ren et al., 2020), macrophages (Niu et al., 2016; Sharma et al., 2018), and adipose mesenchymal stem cells (Liu et al., 2016). EVs are known to contain and carry various bioactive molecules, including proteins, lipids, and nucleic acids (Berezin and Berezin, 2020; Xing et al., 2020). It has been well documented that these exosomal molecules are involved in executing the effects of EVs on EC-to-VSMC regulation. For example, EVs derived from human umbilical vein ECs can modulate VSMCs phenotype via EV-containing microRNAs (Hergenreider et al., 2012). Hyperglycemia stimulated vascular ECs to release exosomes, which transfers a circular RNA (cirrcRNA-0077930) to VSMCs causing senescence in VSMCs (Wang et al., 2020). In addition to non-coding RNAs, exosomal protein Notch3 from high glucose-stimulated ECs was also demonstrated to control VSMC calcification and aging (Lin et al., 2019).

NLR family pyrin domain-containing 3 (NLRP3) inflammasome is an intracellular multimeric protein complex consisting of components including NLRP3, apoptosis-associated speck-like protein (ASC), and pro-caspase-1. Once activated in the cytoplasm, these NLRP3 inflammasome components are aggregated and assembled to form a high-molecular-weight protein complex that triggers cleavage of pro-caspase-1 to the active caspase-1. The caspase-1 activity subsequently converts its substrates such as interleukin-1β (IL-1β) and interleukin-18 (IL-18) to their bioactive forms. Our previous studies have demonstrated that atherogenic stimulation of endothelial NLRP3 inflammasomes contributes the endothelial dysfunction and injury and carotid atherosclerotic lesion formation (Zhang et al., 2015; Wang et al., 2016; Yuan et al., 2018b; Xing et al., 2019; Zhang et al., 2019). Interestingly, the NLRP3 inflammasome products including IL-1β are shown to be secreted out of cells from a pathway that is distinct from the conventional ER-Golgi route of transport and secretion (Lopez-Castejon and Brough, 2011; Daniels and Brough, 2017). Accumulating evidence indicates that EVs, in particular exosomes, are involved in the release of NLRP3 inflammasome products including IL-1β in mammalian cells (Hong et al., 2019; Yuan et al., 2019b). However, it remains undermined whether or not atherogenic stimulation could incite secretion of endothelial-derived NLRP3 inflammasome products through exosome machinery, and thereby these EC-derived exosomes directly promote VSMC phenotype transition.

Exosomes are nanosized membrane vesicles released by fusion of the multivesicular body (MVB), an organelle of the endocytic pathway, with the plasma membrane (Hessvik and Llorente, 2018). MVB-based exosome release is finely controlled by lysosome trafficking and associated with the autophagic pathway due to lysosome fusion with mature MVBs to degrade their contents via autophagy (Davies et al., 2009; Huber and Teis, 2016; Buratta et al., 2020). Indeed, lysosome dysfunction or injury by alkaline agent chloroquine or lysosomal v-ATPase inhibitor bafilomycin A increased the secretion of exosomes in different cells including neurons, epithelial cells, and vascular cells (Akyurek et al., 2000; Price et al., 2001; Piccoli et al., 2011). It has been reported that neutral sphingomyelinase-mediated sphingolipids (ceramide and S1P) participate in exosome biogenesis, sorting intraluminal vesicles (ILVs) into multivesicular bodies (MVBs), membrane invagination or budding of exosome into MVBs, or MVB fusion to membrane for release of ILVs as exosomes (Huber and Teis, 2016). These findings suggest that sphingolipids produced by neutral sphingomyelinase may be primarily involved in exosome biogenesis and fusion with plasma membrane. MVBs can also fuse with and deliver content to lysosomes for degradation. Therefore, the lysosome function can determine the fate of MVBs (i.e., secreted vs. disposed). Indeed, lysosomal sphingolipids are classical regulators of lysosome function and therefore implicated in the control of MVB fate. The lysosomal acid sphingomyelinase (ASM) hydrolyze sphingomyelin into ceramide, which is further converted to sphingosine by lysosomal acid ceramidase (*AC*). It has been shown that abnormal lysosome sphingolipid signaling is associated impaired lysosome function in ECs and VSMCs, which may lead to dysregulation of autophagic flux and enhanced exosome secretion (Zhang et al., 2014; Kapustin et al., 2015; Serban et al., 2016). Our recent studies demonstrated that *AC* gene deletion enhanced the release of exosomes in the coronary arterial ECs (Yuan et al., 2019b) and podocytes (Hong et al., 2019). These data suggest that lysosomeal ceramide accumulation induced by AC inhibition may impair lysosome function and thereby inhibit lysosome-mediated MVB degradation leading to enhanced exosome secretion.

The present study aimed to test the hypothesis that under atherogenic stimulation, ECs secrete NLRP3 inflammasome products such as IL-1β in EVs, and these IL-1β-containing EVs trigger or promote synthetic phenotype transition of VSMCs. Our *in vitro* studies demonstrated that EVs isolated from unstimulated ECs inhibited VSMC proliferation and migration. In contrast, atherogenic stimulation of ECs by oxysterol 7-ketocholesterol (7-Ket) induced NLRP3 inflammasome formation and activation, reduced MVB-lysosome fusion and increased secretion of IL-1β-containing EVs that promoted VSMC proliferation and migration. Moreover, we found that lysosome inhibition by bafilomycin or *AC* gene deletion exaggerated 7-Ket-induced release of IL-1β-containing EVs in ECs. In animal studies using endothelium-specific *AC* gene knockout mice (Asah1^fl/fl^/EC^Cre^) and their WT/WT littermates, we observed that endothelial *AC* deficiency enhanced the secretion of IL-1β-containing EVs by hypercholesterolemic diet treatment in the arterial wall, which was accompanied by the enhanced VSMC synthetic phenotype transition and accelerated neointimal formation. Our findings provide novel mechanistic insights into understanding the pathogenic role of endothelial NLRP3 inflammasome in vascular injury through EVs-mediated EC-to-VSMC regulation.

## Materials and Methods

### Mice

*Asah1*^fl/fl^/EC^cre^ [Cre transgenic mice are from B6.CgTg (Tek-cre) 1Ywa/J 008863)”] and wild-type (WT/WT) mice were generated and characterizatied as previously described (Beckmann et al., 2018; Yuan et al., 2019b). WT/WT and *Asah1*^fl/fl^/EC^cre^ male and female mice (12–20 weeks old) were used for the present study. They were bred and maintained in an environmentally controlled animal facility center (25°C and 40∼50% humidity) with a 12 h light/dark cycle. WT/WT and *Asah1*^fl/fl^/EC^cre^ mice were randomly divided into four groups and fed a Western diet (WD) or normal diet (ND) for 4 weeks. Then these mice were used for partial ligated carotid artery (PLCA) model following by feeding the mice with WD or ND for additional 3 weeks. At the end of treatment, mice were sacrificed, and the blood was collected for EVs analysis, and the carotid arteries were harvested for HE staining and immunocytochemical analysis. All procedures were carried out following the National Institutes of Health guidelines for the care and use of laboratory animals. All animal protocols were approved by the Institutional Animal Care and Use Committee (IACUC) at Virginia Commonwealth University.

### PLCA Model

PLCA was carried out as we described previously (Xia et al., 2014; Yuan et al., 2019b). Briefly, 2% isoflurane was used to anesthetize mice during surgery. The neck of the mouse was sterilized with betadine solution with 5% povidone-iodine. A sterile drape was used to cover the area. A midline incision (1–2 cm) was made to expose the left carotid artery. The carotid arteries were tightly ligated with 6.0 silk suture (external, internal, and occipital) except the superior thyroid artery for blood circulation. The incision was closed with a 5.0 silk suture and disinfected with a betadine solution. After 3 weeks, half of the arteries were isolated and frozen in liquid nitrogen for immunofluorescence staining. Another half of the arteries were stocked in 10% formalin to prepare wax slides for HE staining and immunohistochemistry staining.

### Isolation and Culture of ECs From the Mouse Carotid Artery

Isolation of mouse carotid arterial ECs was performed and characterized as previously described (Li et al., 2013; Kobayashi et al., 2005). ECs were primed with a low dose of LPS (1 ng/ml) for 3 h before any experiments. For the proatherogenic stimulation, cells were treated with 7-Ket (0–10 μg/ml) and then incubated for 21 h. In the case of inhibitors used, the cells were pretreated with carmofur (2 μM), rapamycin (Rap) (10 nM) for 30 min.

### Isolation and Culture of Mouse VSMCs

Mouse VSMC isolation has been previously described (Adhikari et al., 2015; Lu et al., 2019). Briefly, mice were anesthetized with 2% isoflurane. The carotid arteries were then removed and put into PBS on ice. The adventitia was separated from the media layer of the artery under the microscope. The tissue was washed three times with PBS and cut into tiny pieces. The small pieces were washed three times with cell culture medium and then added into a cell culture dish without the medium. After 2 h, fresh medium (FBS (10%) and DMEM supplemented with 2% antibiotics) was then added into the culture dish. The tissues were incubated in a humidified 37°C, 5% CO_2_ incubator. After 5–10 days, VSMCs were isolated when they grew out of the dissected tissue, and VSMCs were cloned by the selection of those cells from cell-growing islands in the dish. Passages 4–10 cells were used for all the experiments.

### Western Blot Analysis

Western blot was used to analyze vimentin and smooth muscle 22α (SM22α) protein expression as described previously (Mo et al., 2018). ECs were collected and homogenized in lysis buffer RIPA Lysis and Extraction Buffer (Thermo Scientific, 89900) for 30 min on ice. After centrifuge, the protein was measured using Bio-Rad Protein Assay Dye (Bio-rad,500006, United States). All samples were normalized to 1 μg/ml. About 20 μg of protein was loaded into the wells of a 12% SDS-PAGE gel and electrophoresis was conducted for 2–3 h at a voltage of 100 V. Protein was transferred to nitrocellulose membranes (Millipore, IPVH00110, United States) and run at a voltage of 100 V for 1 h in the cold room. After being blocked in 5% non-fat milk (Bio-Rad, 1706404, United States) in Tris-buffered saline with Tween-20 (TBST) buffer for 1 h at room temperature, the blot was incubated with primary antibodies overnight at 4°C. The following antibodies were used for immunoblotting: vimentin (1:5,000, Abcam, Cambridge, MA), SM22α (1:5,000, Abcam Cambridge, MA) PCNA (1:5000, Abcam, Cambridge, MA) and a-SMA (1:8000, Abcam, Cambridge, MA). The membrane was incubated in a secondary antibody labeled with HRP for another 1 h at room temperature. The target bands were detected using Odyssey FC Imaging. Anti-β-actin antibody (1:20,000 dilution, Santa Cruz, United States) as a loading control was used to probe this housekeeping gene expression. Image J 6.0 (NIH, Bethesda, MD, United States) or Odyssey software was used to quantify the intensity of the specific proteins.

### Immunofluorescence Staining

The carotid sections or ECs cultured on cover slides were fixed in 4% paraformaldehyde (PFA) for 10–15 min on ice. After being washed 3 times with PBS, the samples were incubated for 2 h or overnight at 4°C with the primary antibodies against following proteins: lysosome marker, anti-lamp-1 antibody (1:500, Abcam, ab25245); EVs marker, anti-CD63 antibody (1:100, Santa Cruz, sc-15363) or MVB marker (Wartosch et al., 2015) anti-VPS16 antibody (1:200, Abcam, ab172654); NLRP3 inflammasome product, anti-IL-1β antibody (1:200, BD, AF-401-NA); inflammasome component, anti-NLRP3 (1:200, Abcam, ab4207), anti-ASC (1:100, Santa Cruz, sc-22514), and anti-procaspase-1 (1:100, Santa Cruz, sc-56036). After being washed 3 times with PBS, samples were further incubated with a second antibody labeled with either Alexa-488- or Alexa-555 for 1 h at room temperature in the darkroom. A confocal laser scanning microscope (Nikon Eclipse Ti confocal microscope, NY, United States) (Fluoview FV1000; Olympus, Tokyo, Japan) was used to take pictures and images were processed using NIS Element imaging software. The fluorescence intensity of the cells or tissues was measured and analyzed with Image J 6.0 (NIH, Bethesda, MD, United States). The colocalization was detected the double staining and measured with Image-Pro Plus version 6.0 software (Media Cybernetics, Bethesda, MD). Pearson correlation coefficient (PCC) was used to show the colocalization of different proteins as described previously (Chen et al., 2018).

### Immunohistochemistry (IHC)

The paraffin sections were heated for 10 min at 65°C. Deparaffinization was performed twice in 100% xylene for 10 min. Hydration was carried out in a series of graded ethanol (100%, 95%, 75%) for 5 min at room temperature. Next, 10 mM of sodium citrate buffer (pH 6.0) was used to retrieve the antigen at over 95°C for 15 min. 3% H_2_O_2_ in methanol was used to quench the endogenous peroxidase activity. Non-specific proteins were blocked with 2.5% horse serum for 1 h at room temperature. The sections were incubated with primary antibodies for 2 h or overnight at 4°C: anti-vimentin (1:5,000, Abcam, Cambridge, MA). The sections were incubated with biotinylated secondary antibodies for 20 min and developed with 3,3’-Diaminobenzidine (DAB) solution for 5 min. Finally, the sections were counterstained in hematoxylin (Sigma, 51275, United States) for 5 min, dehydrated in graded ethanol (75, 95, and 100%), and mounted with permount medium (Fisher scientific, SP15-100). Negative controls were prepared without the primary antibodies. The area percentage of the positive staining was calculated in Image-Pro Plus 6.0 software.

### Morphologic Examination and Medial Thickening Analysis

HE staining of carotid sections was used to study the morphological changes as described previously (Chen et al., 2015). Briefly, the carotid was perfused with cold PBS for 5 min and 4% cold PFA for another 5 min. Then the carotid was isolated and immersed into 10% neutral buffered formalin. Next, the formalin-fixed carotid was embedded in paraffin and then cut into 7 μm serial sections for histopathological evaluation. For HE staining, the sections were heated for 10 min at 65°C and deparaffinization was performed twice in 100% xylene for 10 min. The samples were rehydrated with 100, 95, and 75% ethanol to water and immersed in hematoxylin and hydrochloride alcohol. Once the color turned to blue, the sections were stained with eosin. After that, the sections were rinsed with running water and dehydrated with different grades of ethanol. Finally, Dibutyl phthalate Polystyrene Xylene (DPX) was used to mount the slides. Intimal-medium thickening of carotid arteries was examined using Image-Pro Plus 6.0 software (Media Cybernetics Inc., United States).

### Isolation of Extracellular Vesicles

To purify the EVs, we used differential ultracentrifugation as described previously (Kapustin et al., 2015). Briefly, cell culture medium or plasma from mice was collected and centrifuged at 300 g at 4°C for 10 min to remove detached cells or debris. The supernatant was collected and filtered through 0.22 μm filters to remove contaminating apoptotic bodies, microvesicles, and cell debris. EVs were spun down by ultracentrifugation of the supernatant at 100, 000 g for 90 min at 4°C (Beckman 70.1 T1 ultracentrifuge rotator), washed in ice-cold filtered PBS and resuspended in 50 μl ice-cold filtered PBS. These EV samples are ready for use or stored at −80°C. For NanoSight microparticle analysis, the samples are diluted into filtered PBS.

### Nanoparticle Tracking Analysis

Nanoparticle Tracking Analysis (NTA) was used to characterize EVs with the light scattering mode of the NanoSight LM10 (NanoSight Ltd., Amesbury, United Kingdom). Five frames (30 s each) were captured for each sample with background level 10, camera level 12, and shutter speed 30. Captured EVs 3D distribution images were analyzed using NTA software (Version 3.2 Build 16). Particle sizes ranged between 40-150 nm were calculated.

### Measurement of IL-1β Secretion

The culture medium or purified EVs were collected for IL-1β quantification with an IL-1β ELISA kit according to the manufacturer’s instructions (Li et al., 2014). In brief, 1 ml of the culture medium or 50 μl of lysed EVs was added to a microplate strip well and incubated for 2 h at room temperature. Then, the solution was mixed with IL-1β conjugate and incubated for another 2 h at room temperature. 5 times washing was performed between and after the two incubations. 100 μl of substrate solution was applied to generate chemiluminescence. Chemiluminescent absorbance was determined using a microplate reader at 450 nm. The IL-1β level was quantified by relating the sample readings to the generated standard curve.

### Scratch Wound-Healing Assay

Scratch wound-healing assay was used to assess the VSMC migration as described (Liang et al., 2007). In brief, 1.5 × 10^6^ of VSMCs were cultured in a 3.5 cm cell culture dish for up to 2 days till 100% confluency. The VSMC monolayer was scratched off with a 200 ul sterile pipette tip and the detached cells were washed away with PBS. The VSMCs were cultured in DMEM containing 2% FBS for 24 h and imaged by an inverted microscope. The VSMC migration ability was evaluated by the percentage of wound-healing (migrated cell area in the wounded region/initial area of wounded region × 100%).

### Cell Proliferation Assay

The WST-1 Kit was used to measure VSMC proliferation. The VSMCs were cultured at 0.5 × 10^4^ cells in 24-well plates containing 10% FBS supplemented DMEM overnight. On the next day, VSMCs were replaced with fresh DMEM supplemented 2% FBS. On day 5, the proliferation of VSMCs was examined using WST-Kit. Briefly, the medium was discarded and incubated for 4–6 h with 200 μl of DMEM with WST-1 in the 37°C incubator. The medium was transferred to a 96-well plate to measure the absorbance (OD Value) at 450 nm. The data were expressed as ratios of the control value.

### Statistics

Data were shown as means ± standard error (SE). Values were analyzed for significant differences between and within multiple groups using ANOVA for repeated measures, followed by Duncan’s multiple range test. Significant differences between the two groups of experiments were examined using the Student’s *t*-test. The statistical analysis was performed with SigmaPlot 12.5 software (Systat Software, San Jose, CA, United States). Statistical significance was defined when *P* < 0.05.

## Results

### Extracellular Vesicles Isolated From ECs Changed VSMCs Phenotype From Contractile to the Synthetic Status

**FIGURE 1:**
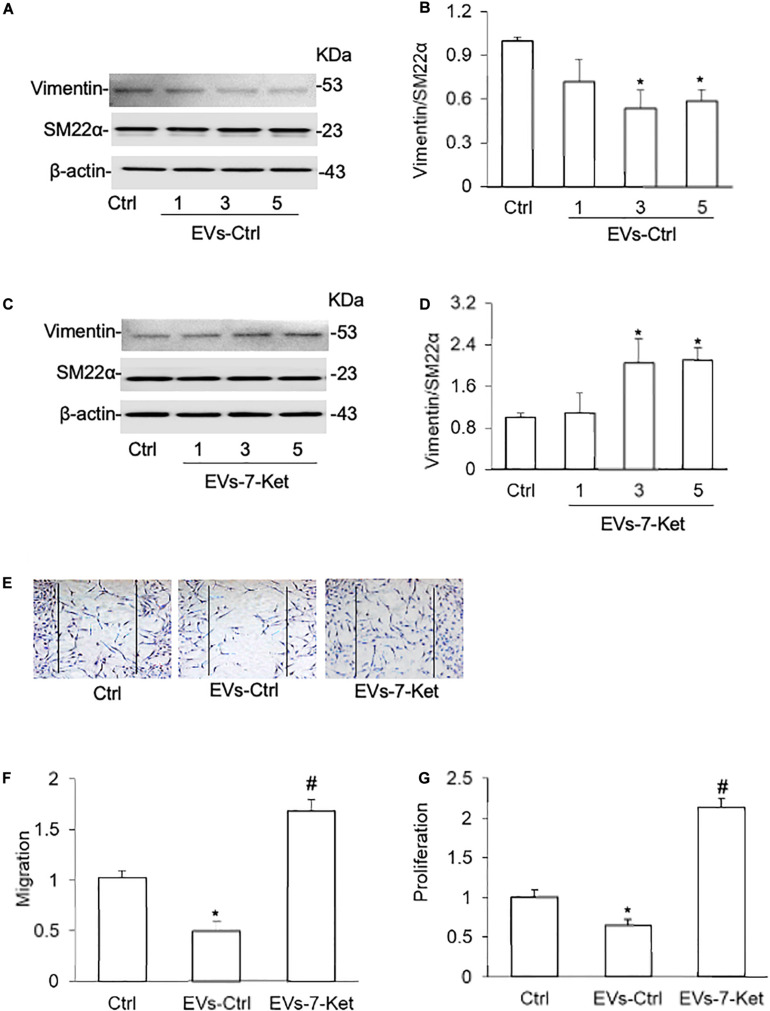
Phenotype transition of vascular smooth muscle cells (VSMCs) cocultured with extracellular vesicles (EVs) isolated from the primary cultured carotid arterial endothelial cells (ECs). Primary cultured carotid arterial ECs were treated with 7-Ketocholesterol (7-Ket) (0-10 μg/ml) for 24 h. **(A,C)** Representative Western blot gel documents showing the expression of vimentin and SM22α induced by EVs collected from the carotid arterial ECs with (EVs-7-Ket) or without 7-Ket treatment (EVs-Ctrl). **(B,D)** The summarized data showing the ratio of vimentin with SM22α protein. **(E)** Representative wound healing assay images presenting the (1,3,5 × 10^9^ EVs) effects of EVs on CAMs migration. **(F)** Summarized data showing the dose effects of EVs on CAMs migration. **(G)** Summarized data showing the dose effects of EVs on CAMs proliferation. Data are expressed as means ± SEM, *n* = 5. *p < 0.05 vs. Ctrl group.

We first examined whether EC-derived EVs act on VSMCs phenotype transition. EVs were isolated from mouse primary cultured carotid ECs with or without 7-Ket treatment and then co-cultured with VSMCs. VSMCs were treated with 7-Ket at concentration of 2–10 μg/ml. This dose was chosen to avoid cell death caused by higher concentration of 7-Ket (50 μg/ml or above). It was found that EVs isolated control ECs (EVs-Ctrl) dose-dependently decreased the vimentin and proliferating cell nuclear antigen (PCNA) expression in VSMCs (Figures 1A,B and Supplementary Figures 1A,B), whereas EVs from 7-Ket-treated ECs (EVs-7-Ket) significantly increased the expression of synthetic phenotype marker vimentin in VSMCs (Figures 1C,D and Supplementary Figures 1D,E). In contrast, ECs (EVs-Ctrl) dose-dependently increased the α-SMA expression (Supplementary Figures 1A,C), whereas EVs from 7-Ket-treated ECs (EVs-7-Ket) significantly decreased α-SMA expression in VSMCs (Supplementary Figures 1D–F). However, none of these EVs had any effects on the expression of contractile phenotype marker SM22α (Figures 1A,D). Moreover, the EVs from control ECs significantly reduced VSMC migration (Figures 1E,F) and proliferation (Figure 1G). However, the EVs from 7-Ket-treated ECs showed the opposite effects. It should be noted that in wound-healing migration assay, VSMCs were cultured in a low-serum medium (2% FBS) to minimize the effects of proliferation on cell migration. Together, these results suggest that under different conditions, EC-derived EVs have distinct effects on the VSMC phenotype transition.

### 7-Ket Stimulated NLRP3 Inflammasome Formation and Activation in ECs

**FIGURE 2:**
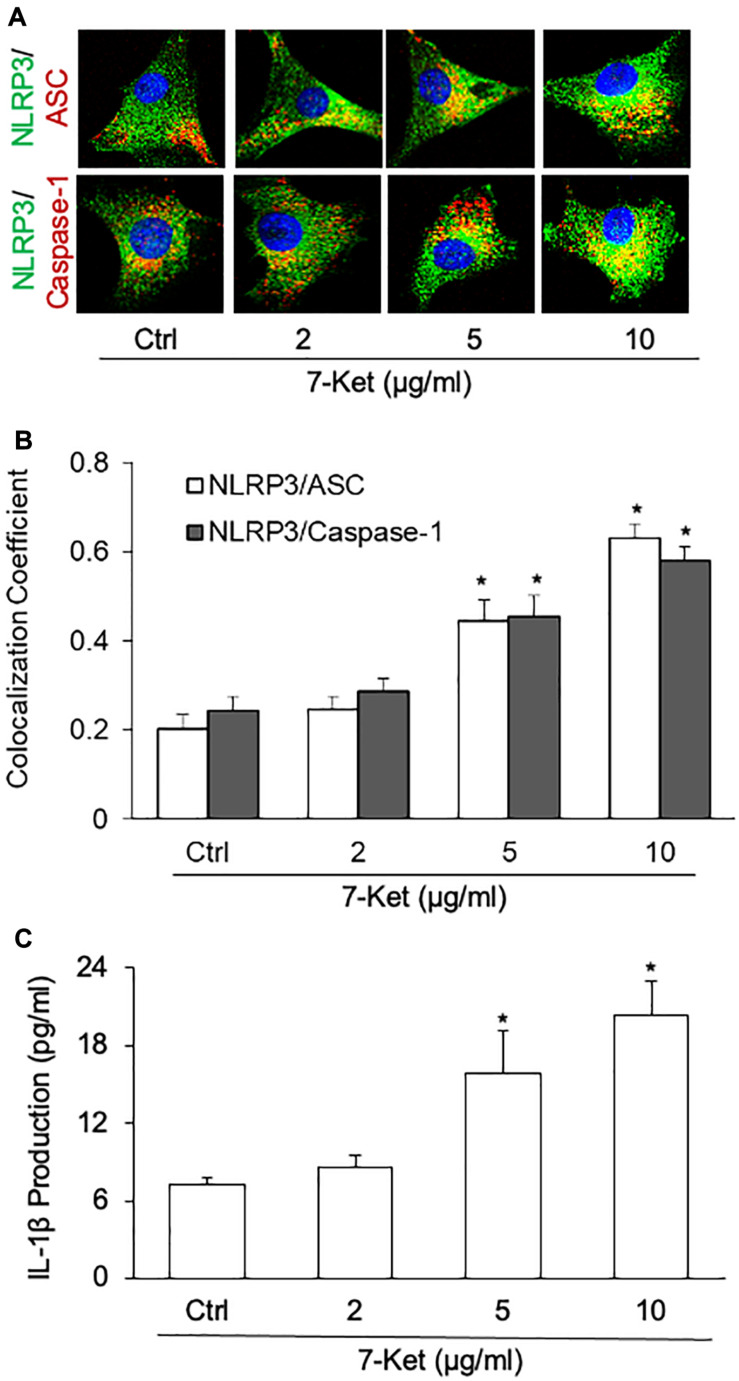
NLRP3 inflammasome formation and activation dose-dependently stimulated by 7-Ket in the primary cultured carotid arterial ECs. **(A)** Representative fluorescent confocal microscope images displaying the yellow dots or patches showing the colocalization of NLRP3 (green) with ASC or caspase-1 (Red). **(B)** The summarized data (*n* = 5) showing the colocalization coefficient of NLRP3 with ASC or caspase-1. **(C)** The summarized data (*n* = 6) showing IL-1β secretion. Data are expressed as means ± SEM. *p < 0.05 *vs.* Ctrl group.

The oxysterol 7-Ket was reported to stimulate NLRP3 inflammasome activation and formation in ECs (Shi et al., 2015; Koka et al., 2017; Yuan et al., 2018b), VSMCs (Yuan et al., 2018a; Chen et al., 2019), macrophage cells (Li et al., 2014; Calle et al., 2019), leading to the release of proinflammatory cytokines such as IL-1β and IL-18. Using confocal immunofluorescent staining, we confirmed that 7-Ket dose-dependently increased the colocalization of NLRP3 with ASC or caspase-1 in the primary cultured ECs (Figures 2A,B). Consistently, 7-Ket increased the release of IL-1β in the primary cultured ECs (Figure 2C). Therefore, these results suggest that 7-Ket stimulates NLRP3 inflammasome formation and activation as well as IL-1β release in ECs.

### 7-Ket Induced the Release of IL-1β-Containing Extracellular Vesicles in ECs

**FIGURE 3:**
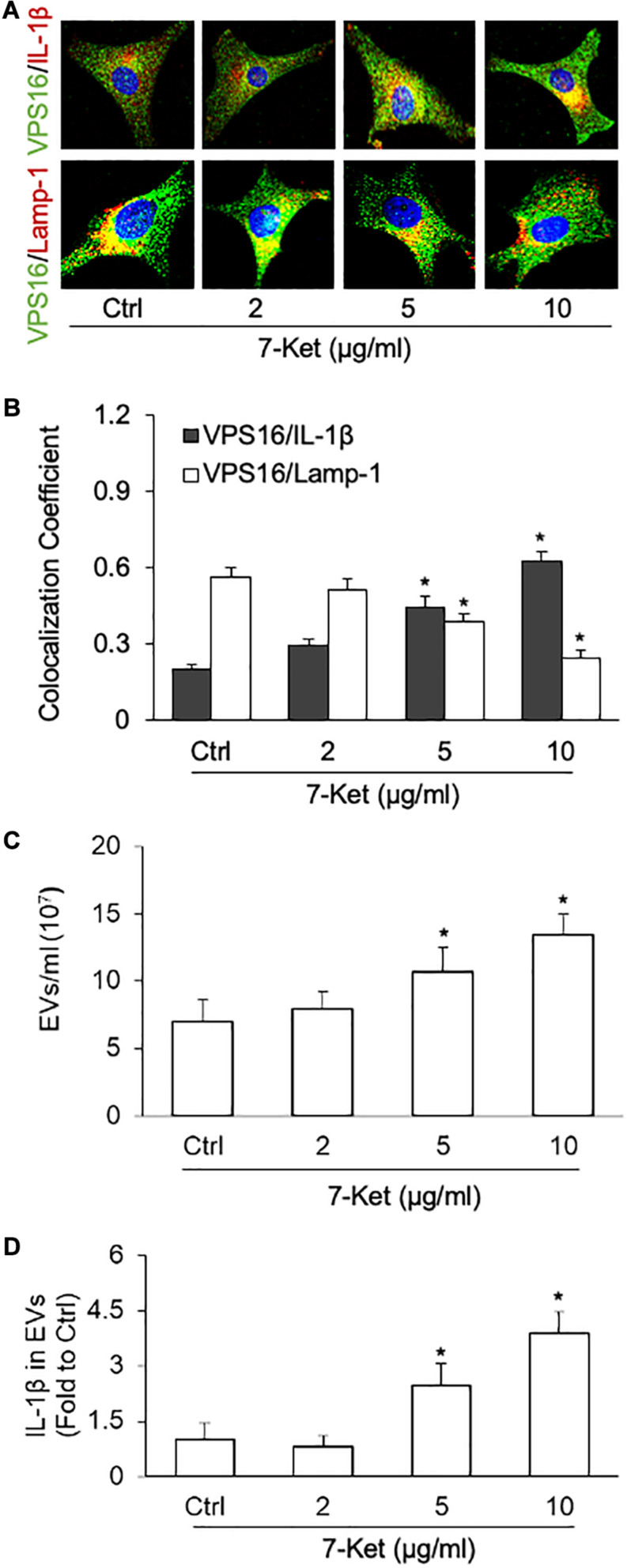
NLRP3 inflammasome-dependent IL-1β secretion dose-dependently induced by 7-Ket *via* EVs in the primary cultured carotid arterial ECs. **(A)** Representative fluorescent confocal microscope images showing the colocalization of VPS16 (green) with IL-1β or Lamp-1 (Red). **(B)** The summarized data showing the colocalization coefficient of VPS16 with IL-1β or Lamp-1. **(C)** The summarized data showing the EVs released into the cell culture medium as measured by nanoparticle tracking analysis (NTA) using the NanoSight NS300 nanoparticle analyzer (50–150 nm). **(D)** The summarized data showing IL-1β products in the EVs isolated from ECs measured by ELISA Kit. Data are expressed as means ± SEM (*n* = 5). *p < 0.05 vs. Ctrl group.

Previous studies have demonstrated that the NLRP3 activators [calcium oxalate and monosodium urate (MSU) crystals, ATP, β-glucans, viral RNA], as well as caspase-4-dependent activation, induce secretion of EVs and EV-associated proteins (Cypryk et al., 2018). Here, using immunofluorescence confocal microscopy, we observed that 7-Ket treatment increased the interaction of MVB with IL-1β as shown by increased colocalization between MVB marker VPS16 with IL-1β (Figures 3A,B). In contrast, 7-Ket decreased the interaction of MVBs with lysosomes as shown by decreased colocalization of VPS16 with lysosome marker Lamp1 (Figures 3A,B) (Supplementary Figures 3A,B). Quantification of EVs by NTA revealed that 7-Ket increased the number of EVs (in size of 50–150 nm) released by ECs (Figure 3C). Moreover, after normalizing the IL-1β content over the number of EVs, we found that 7-Ket also increased the IL-1β level per EV (Figure 3D). These results suggest that 7-Ket not only increases the number of IL-1β-containing EVs released by ECs, but also increases the relative IL-1β content in these EVs.

### Role of Lysosomal AC in the Secretion of IL-1β-Containing Extracellular Vesicles in ECs

**FIGURE 4:**
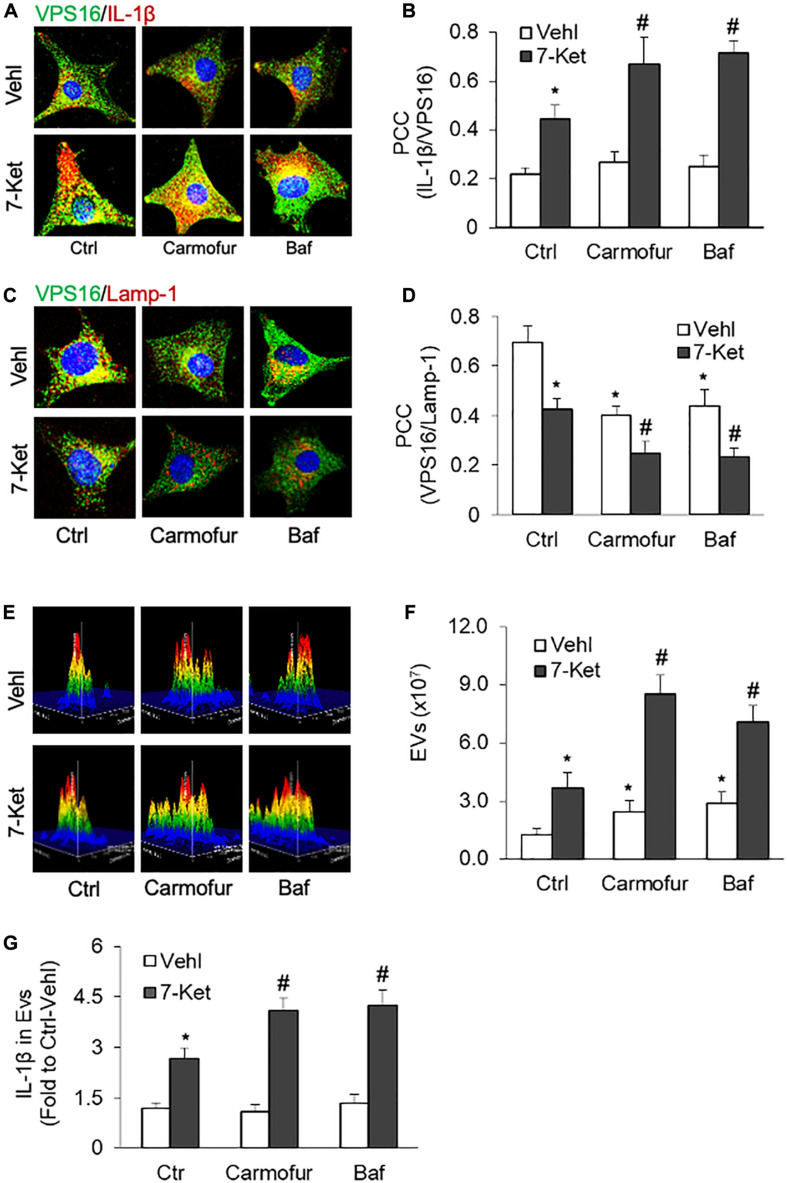
Effects of acid ceramidase (*AC*) and lysosome on the EVs release with IL-1β in the carotid arterial ECs. Primary cultured ECs were treated with *AC* inhibitor carmofur (2 μM) or lysosome inhibitor bafilomycin (10 nM) for 2 h before being treated with 10 μg/ml of 7-Ket for another 24 h. **(A)** Representative fluorescent confocal microscope images showing the colocalization of VPS16 (green) with IL-1β (Red). **(B)** The summarized data showing the colocalization coefficient of VPS16 with IL-1β. **(C)** Representative fluorescent confocal microscope images showing the colocalization of VPS16 (green) with Lamp-1 (Red). **(D)** The summarized data showing the colocalization coefficient of VPS16 with Lamp-1. **(E)** Representative 3D histograms showing the secretion of EVs in the cell culture medium as measured by nanoparticle tracking analysis (NTA) using the NanoSight NS300 nanoparticle analyzer. **(F)** The summarized data showing the released EVs from the cell culture medium (50–150 nm). **(G)** The summarized data showing the secretion of IL-1β via EVs. Data are expressed as means ± SEM, *n* = 5. *p < 0.05 *vs.* Vehl-Ctrl group; #*p* < 0.05 vs. 7-Ket-Ctrl group.

Recent studies have demonstrated that the lysosomal *AC* plays a critical role in regulating lysosome trafficking (Park and Schuchman, 2006; Laurier-Laurin et al., 2014), and its interaction with MVBs, an event determines the fate of MVBs and thereby EVs release (Eitan et al., 2016). We next examined whether inhibition of *AC* or lysosome function could enhance 7-Ket-induced secretion of IL-1β-containing EVs in ECs. In this regard, the ECs were treated with an *AC* inhibitor carmofur or a lysosome inhibitor bafilomycin A. As shown in Figures 4A,B and Supplementary Figure 4, the colocalization of VPS16 with IL-1β by 7-Ket was further enhanced in ECs treated with camofur or bafilomycin. In parallel, the colocalization of VPS16 with Lamp-1 by 7-Ket was further significantly attenuated in ECs by camofur or bafilomycin (Figures 4C,D and Supplementary Figure 5). Consistently, both carmofur and bafilomycin enhanced 7-Ket-induced increases in the release of EVs (Figures 4E,F) and the IL-1β levels in isolated EVs (Figure 4G).

**FIGURE 5:**
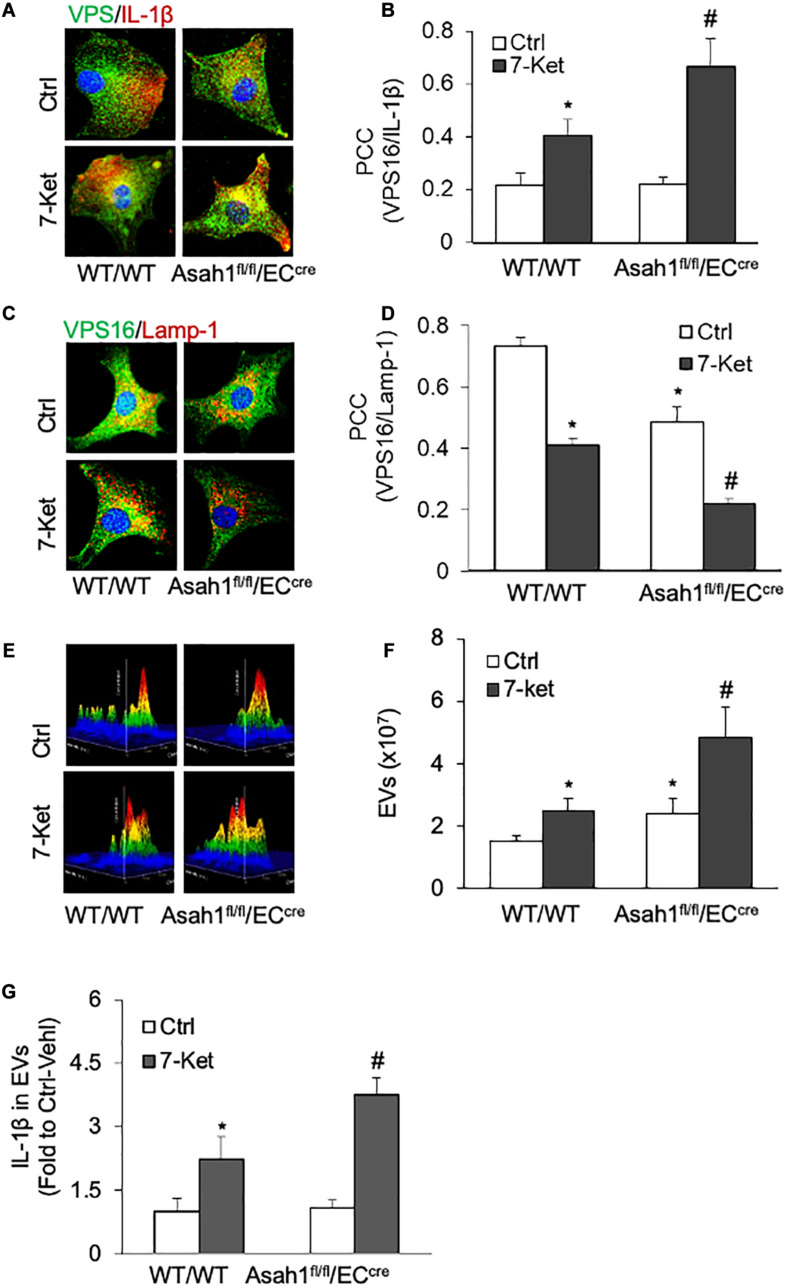
Effects of *AC* deletion on the release of IL-1β *via* EVs in the carotid arterial ECs. **(A)** Representative fluorescent confocal microscope images showing the colocalization of VPS16 (green) with IL-1β (Red). **(B)** The summarized data showing the colocalization coefficient of VPS16 with IL-1β. **(C)** Representative fluorescent confocal microscope images showing the colocalization of VPS16 (green) with Lamp-1 (Red). **(D)** The summarized data showing the colocalization coefficient of VPS16 with Lamp-1. **(E)** Representative 3D histograms showing the secretion of EVs in the cell culture medium as measured by nanoparticle tracking analysis (NTA) using the NanoSight NS300 nanoparticle analyzer. **(F)** The summarized data showing the released EVs from the cell culture medium (50–150 nm). **(G)** The summarized data showing the secretion of IL-1β via EVs. Data are expressed as means ± SEM, *n* = 5. *p < 0.05 vs. WT/WT-Ctrl group; #*p* < 0.05 vs. WT/WT-7-Ket group.

The role of *AC* in the secretion of IL-1β-containing EVs was further investigated in *AC*-deficient ECs isolated from EC-specific *AC* knockout (*Asah1*^fl/fl^/EC^cre^) mice. As shown in Figures 5A–G, 7-Ket-induced increase in the colocalization of VPS16 with IL-1β was further augmented by *AC* gene deficiency (Figures 5A,B), whereas a 7-Ket-induced decrease in the colocalization of VPS16 with Lamp-1 was further attenuated in *AC*-deficient ECs (Figures 5C,D). Moreover, *AC* gene deficiency augmented 7-Ket-induced increases in EV release (Figures 5E,F) and exosomal IL-1β levels (Figure 5G). Taken together, these findings from pharmacological intervention and genetic approaches suggest that lysosomal *AC* signaling plays a critical role in governing the secretion of IL-1β through the EV pathway in ECs.

### Endothelial AC Gene Deletion Enhanced IL-1β Secretion via Extracellular Vesicles in the Carotid Arterial Endothelium

**FIGURE 6:**
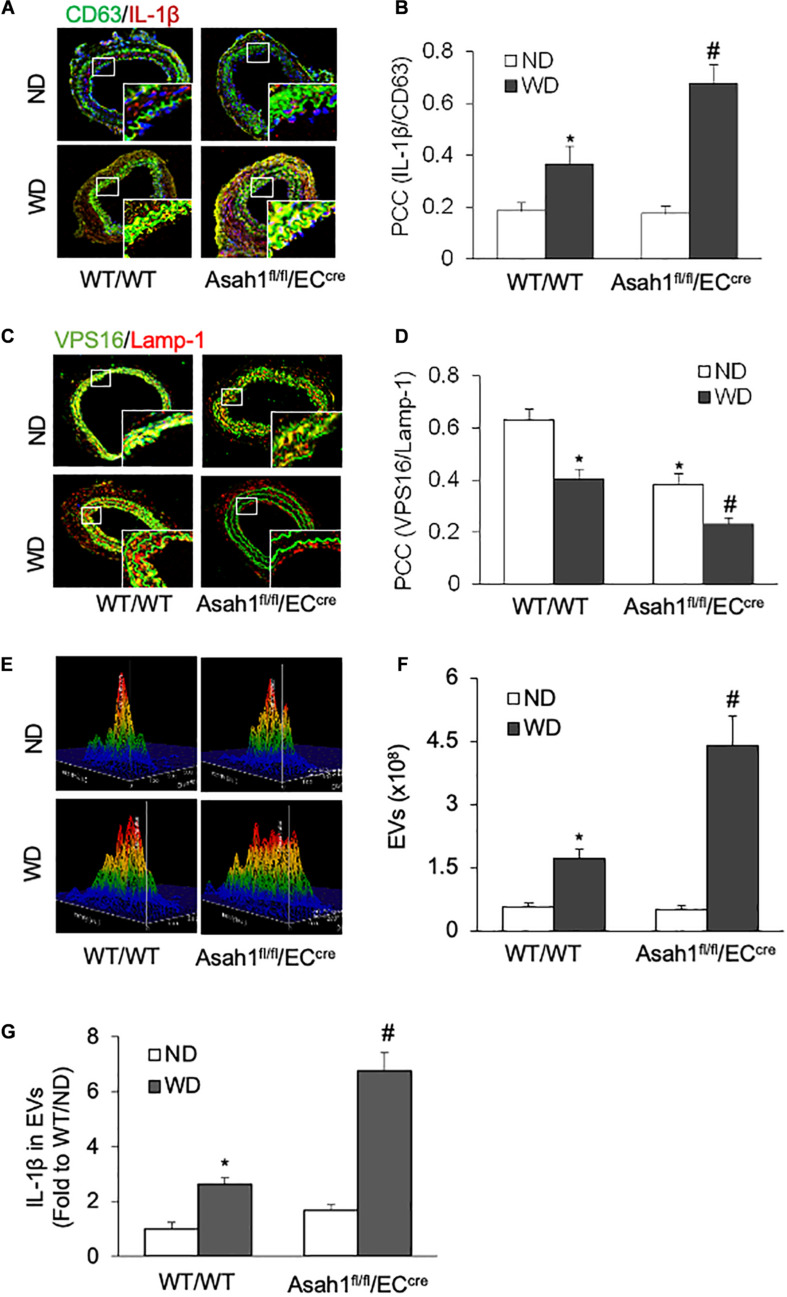
Effects of endothelial AC deficiency on the EV secretion in the carotid arterial wall of mice. **(A)** Representative fluorescent confocal microscope images displaying the yellow dots or patches showing the colocalization of CD63 (green) with IL-1β (Red). **(B)** The summarized data showing the colocalization coefficient of CD63 with IL-1β. **(C)** Representative fluorescent confocal microscope images showing the colocalization of VPS16 (green) with Lamp-1 (Red). **(D)** The summarized data showing the colocalization coefficient of VPS16 with Lamp-1. **(E)** Representative 3D histograms showing the secretion of EVs in plasma as measured by nanoparticle tracking analysis (NTA) using the NanoSight NS300 nanoparticle analyzer. **(F)** The summarized data showing the secretion of EVs in the plasma (50–150 nm). **(G)** The summarized data showing the IL-1β secretion in the EVs from plasma. Data are expressed as means ± SEM, *n* = 5. *p < 0.05 vs. WT/WT-ND group; #*p* < 0.05 vs. WT/WT-WD group.

We next examined whether or not hypercholesterolemia increases the release of IL-1β-containing EVs from the arterial endothelium *in vivo*. To introduce neointimal injury in carotid arteries, we established a mouse PLCA model in WT/WT and EC-specific *AC* gene knockout (*Asah1*^fl/fl^/EC^cre^) mice fed ND or WD. As shown in Figures 6A,B and Supplementary Figure 6A, the confocal microscopic analysis demonstrated that WD treatment increased the colocalization of IL-1β with EV marker CD63 in the arterial wall of WT/WT mice, whereas such effect was further enhanced in *Asah1*^fl/fl^/EC^cre^ mice. Figures 6C,D and Supplementary Figure 6A demonstrated that WD significantly decreased the colocalization of VPS16 with Lamp-1 in the arterial wall of WT/WT mice, which was further reduced in *Asah1*^fl/fl^/EC^cre^ mice. We also found that WD treatment increased the concentration of EVs and exosomal IL-1β levels in the plasma of WT/WT mice, effects further augmented in *Asah1*^fl/fl^/EC^cre^ mice (Figures 6E–G).

### Endothelial AC Deletion Accelerated VSMC Phenotype Transition and Neointima Formation in Partial Ligated Carotid Arteries

**FIGURE 7:**
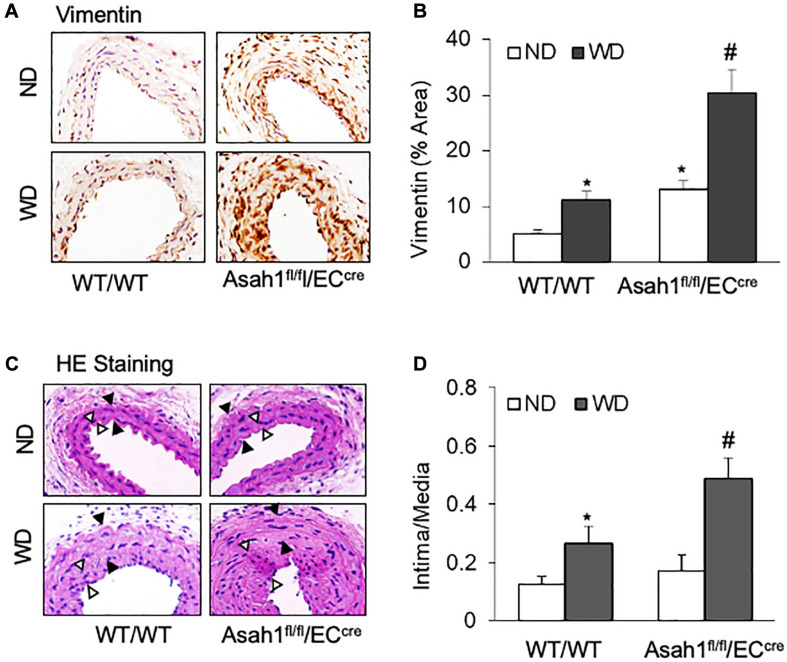
Effects of endothelial *AC* deficiency in the carotid arterial smooth muscle on phenotype changes and neointima formation. **(A)** Representative microscope images of tissue slides with IHC staining showing the expression of vimentin on the mouse carotid arterial wall. **(B)** The summarized data showing the density of vimentin stained with the anti-vimentin antibody. **(C)** HE staining showing the neointima and media on the mouse carotid arterial wall. AOI: the media area (black arrowheads) and the intima area (white arrowheads). **(D)** Quantitative analysis of vascular lesions in PLCA represented by calculation of the ratio between arterial intima and media area. Data are expressed as means ± SEM, *n* = 5. *p < 0.05 vs. WT/WT-ND group; #*p* < 0.05 vs. WT/WT-WD group.

As shown in Figures 7A,B, IHC staining showed that WD treatment increased the vimentin expression in the media region of carotid arteries in WT/WT mice, whereas such WD-induced increases in vimentin expression were more pronounced in *Asah1*^fl/fl^/EC^cre^ mice, particularly in the neointimal region. HE staining (Figure 7C) and quantification analysis of intima-over-media ratio (Figure 7D) demonstrated that WD treatment induced only a mild neointimal injury in the carotid arteries in WT/WT mice, and a more severe neointima formation by WD was observed in *Asah1*^fl/fl^/EC^cre^ mice.

## Discussion

The present study indicated that endothelial NLRP3 inflammasome activation is critically involved in reversing endothelial EV-induced VSMC phenotype transition by hypercholesterolemia stimulation. Our studies demonstrated that unstimulated ECs secreted EVs that prevent the transition of VSMCs toward a synthetic phenotype, whereas 7-Ket-treated ECs released an increased amount of EVs that contain NLRP3 inflammasome product IL-1β and these EVs promote synthetic phenotype transition of VSMCs. Our results further proved that *AC* gene deletion or inhibition significantly enhanced the release of IL-1β-containing EVs, which was accompanied by the accelerated VSMC synthetic phenotype transition and enhanced medial thickening of carotid arteries during hypercholesterolemia.

Vascular smooth muscle cells are a major cell type presented at all stages of an atherosclerotic plaque, and the increased VSMC proliferation is found during early atherogenesis or upon vascular injury (Basatemur et al., 2019). VSMC migration occurs during several critical physiological and pathological processes ranging from vascular development and early remodeling, response to injury as well as cardiovascular diseases (Tahir et al., 2015; Afewerki et al., 2019). EC-VSMC communication is critical for vascular repair and remodeling in the pathological progression of atherogenesis (Bennett et al., 2016; Gimbrone and Garcia-Cardena, 2016). Recent studies showed that EVs carrying proteins, nucleic acids, and lipids shuttle back and forth between ECs and VSMCs or other cells, which operates in a coordinated manner to maintain the vascular homeostasis (Zhao et al., 2017; Kim et al., 2019). In the present study, we found that unstimulated ECs continuously secreted “control” EVs that can reduce expression of vimentin, a dedifferentiation marker, in co-cultured VSMCs and inhibit their proliferation and migration (Figure 1). Conversely, ECs with atherogenic stimulation by 7-Ket released “synthetic” EVs that upregulated vimentin expression and increased proliferation and migration of VSMCs. The distinct biological activity between “control” and “synthetic” EVs may be reflected by different exosomal cargos that modulate on VSMC functions. For example, overexpressing Kruppel-like factor five induced VSMC phenotype by released EVs that are enriched in miR-155 (Zheng et al., 2017). Similarly, expression of Kruppel-like factor 2 or exposure to laminar shear stress enhances release of EC EVs which contained miR-143/145, which also was reported to switch VSMC phenotype via EVs (Vacante et al., 2019). It should be noted that both “control” and “synthetic” EVs did not change the expression of typical contractile protein SM22α in our experimental settings suggesting that only a subset of signaling pathways are modulated in an EV-dependent manner. Collectively, our results demonstrated a reversal of endothelial EV-induced VSMC phenotype transition upon atherogenic stimulation when ECs release “synthetic” EVs instead of “control” EVs.

NLRP3 inflammasome formation and activation initiate the secretion of inflammasome products such as IL-1β. NLRP3 inflammasome activation has been implicated in the initiation or development of different inflammatory diseases such as gout (Kingsbury et al., 2011), myocardial infarction (Jalil and Ghazi, 2020), and diabetes (Wan et al., 2019), obesity (Sokolova et al., 2019), glomerular injury (Li et al., 2017), and atherosclerosis (Zhuang et al., 2019). The inflammatory cytokine IL-1β has been reported to repress expression of multiple SMCs differentiation marker genes and induce expression of proinflammatory genes such as prostaglandin-endoperoxide synthase 2and chemokine (C–C) motif ligand 2 in cultured SMCs (Alexander et al., 2012). Recent studies reported that inflammasome products such as IL-1β could directly affect the VSMC phenotype transition. For example, IL-1β enhanced VSMC proliferation and migration *via* P2Y2 receptor-mediated RAGE expression and HMGB1 release (Eun et al., 2015). IL-1β may also modulate VSMC phenotype to a distinct inflammatory state relative to PDGF-DD via NF-κB-dependent mechanisms (Alexander et al., 2012). The secretion of NLRP3 inflammasome products including IL-1β depends upon the processing of a precursor form following the assembly of the multi-molecular inflammasome complex (Martin-Sanchez et al., 2016; Arriola Benitez et al., 2019). Intriguingly, IL-1β is not secreted through the conventional ER–Golgi route of protein secretion and the precise mechanism of IL-1β release remains unknown. Recent studies highlight the role of exosomes/EVs in mediating the release of NLRP3 inflammasome products such as IL-1β. For example, IL-1β secretion stimulated by P2 × 7 receptors is dependent on inflammasome activation (Karmakar et al., 2016) and correlated with exosome release in murine macrophages (Qu et al., 2007). Cinzia Pizzirani also reported stimulation of plasma membrane receptors for extracellular nucleotides named P2 receptors caused the release of IL-1β-loaded EVs from human dendritic cells (Pizzirani et al., 2007). EVs mediated IL-1β secretion also were found in human THP-1 monocytes and microglial cells (MacKenzie et al., 2001; Bianco et al., 2005). Our recent studies indeed demonstrated that exosomes mediate the release of IL-1β in ECs and podocytes (Hong et al., 2019; Yuan et al., 2019b). However, the pathophysiological action of these NLRP3 inflammasome-related EVs release on VSMCs is not investigated. In the present study, we proved that in ECs, 7-Ket induced the NLRP3 inflammasome assembly and activation followed by increased release of IL-1β (Figure 2). We further revealed that 7-Ket-induced NLRP3 inflammasome activation was accompanied by increased inclusion of IL-1β proteins in MVBs, reduced lysosome-MVB interaction, enhanced EV release, and elevated exosomal IL-1β levels (Figure 3). These results support the view that these IL-1β-containing EVs belong to, at least a subset, of “synthetic” EVs released by ECs during atherogenic stimulation. Because EVs carry a varite of contents such as microRNAs, proteins, and lipids, our studies did not exclude the possibility that these EV contents alone or in combination with IL-1β contribute to the action of “synthetic” EV on VSMCs. The precise mechanism deserves further elucidation.

The exosome secretion is dependent on a dynamic regulation between exosome biogenesis from ILVs in MVBs and lysosome-mediated disposal of MVBs (Chistiakov et al., 2015a; Boulanger et al., 2017; Hafiane and Daskalopoulou, 2018). MVBs can fuse with autophagosomes to form amphisomes that subsequently fuse with lysosomes for their degradation (Fader et al., 2008; Baixauli et al., 2014). Therefore, the lysosome function determines the fate of MVBs and thereby controls exosome release. Indeed, lysosome dysfunction or injury by various lysosomotropic agents such as bafilomycin A and chloroquine are reported to increase the exosome release. Recently, we demonstrated that inhibition of lysosomal *AC* causes the lysosome trafficking dysfunction leading to impaired fusion of lysosomes with MVBs (Li et al., 2019; Yuan et al., 2019b; Bhat et al., 2020). *AC* inhibition blocks the lysosomal TRPML1-Ca^2+^ channel activity and disturbs lysosome-MVB interaction resulting in increased exosome release in podocytes and VSMC (Li et al., 2019; Bhat et al., 2020). Here, we demonstrated that 7-Ket-induced increase in IL-1β inclusion in MVB, decrease in lysosome-MVB interaction, EV release, and elevation of exosomal IL-1β levels were more significantly changed in *AC*-deficient ECs than WT/WT control ECs (Figure 5). These effects of *AC* deficiency were mimicked by *AC* inhibitor carmofur or lysosome inhibitor bafilomycin A (Figure 4). These data indicate that lysosomal *AC* critically controls 7-Ket-induced secretion of IL-1β-containing EVs in ECs.

In our animal studies, we examined whether *AC* deficiency in the endothelium could augment the release of IL-1β-containing EVs into the arterial wall that contribute to hypercholesterolemia-induced vascular remodeling. Our previous studies have demonstrated that in the PLCA model, hypercholesterolemic injury by WD promotes VSMC dedifferentiation into synthetic phenotypes, leading to VSMC proliferation and migration, which are essential to the development of neointima (Yuan et al., 2018a; Yuan et al., 2019a). To this end, we generated endothelium-specific *AC* gene knockout mice (Asah1^fl/fl^/EC^cre^) and used these mice and their WT littermates to produce the PLCA model with ND or WD. It was found that the deficiency of *AC* significantly enhanced WD-induced expression of EV marker CD63 and its association with IL-1β in the carotid arterial wall in Asah1^fl/fl^/EC^cre^ mice compared to those in WT/WT mice. In contrast, the WD-induced more significant reduction of MVB (VPS16) interaction with lysosomes (Lamp1) in Asah1^fl/fl^/EC^cre^ mice compared to WT/WT mice. Importantly, we also found that WD increased the concentration of IL-1β-containing EVs in the plasma of WT/WT mice, and this plasma EV release was further enhanced in Asah1^fl/fl^/EC^cre^. Together, these results support the view that under hypercholesterolemic condition, endothelium secretes IL-1β-containing EVs into the arterial wall to mediate the proximal EC-to-VSMC interaction. In the meantime, these EVs are secreted into plasma possibly for distal communication with other cells. Importantly, our results showed that the augmented secretion of IL-1β-containing EVs in Asah1^fl/fl^/EC^cre^ mice was correlated with higher expression of vimentin and more severe neointimal injury in the carotid arterial wall compared to WT/WT mice. It should be noted that under the control condition (ND), Asah1^fl/fl^/EC^cre^ mice exhibited reduced MVB-lysosome interaction and mildly increased vimentin expression compared to WT/WT mice. However, there was no difference observed in the secretion of IL-1β-containing EVs and neointima formation in these mice. Consistently, *AC* inhibition did not elicit the release of IL-1β-containing EVs in cultured unstimulated ECs. Therefore, these data further indicate that hypercholesterolemic condition is required to produce IL-1β-containing EVs *in vitro* and *in vivo*.

In summary, the present study demonstrated that atherogenic stimulation induces NLRP3 inflammasome activation in ECs and reverses the endothelial EV-induced VSMC phenotypic transition. Mechanistically, unstimulated ECs secrete “control” EVs that tend to prevent VSMC synthetic phenotype transition. However, upon atherogenic stimulation, ECs increasingly release EVs that carry NLRP3 inflammasome products such as IL-1β, and these IL-1β-containing EVs are considered as “synthetic” EVs promoting VSMC synthetic phenotypes, leading to VSMC proliferation and migration, which are essential in the development of neointima. Lysosomal *AC* critically controls the release of IL-1β-containing EVs in the arterial wall during the development of vascular complications associated with hypercholesterolemia. Targeting EV-mediated release of NLRP3 inflammasome products regulated by *AC* may represent a novel strategy for the prevention and treatment of vasculopathy with hypercholesterolemia.

## Data Availability Statement

The raw data supporting the conclusions of this article will be made available by the authors, without undue reservation.

## Ethics Statement

The animal study was reviewed and approved by the Institutional Animal Care and Use Committee (IACUC) at Virginia Commonwealth University.

## Author Contributions

XY participated in research design, conducted the experiments, performed the data analysis, and wrote the manuscript. OB, AS, and AD revised the manuscript. YZ and P-LL participated in the research design, performed the data analysis, and contributed to the writing of the manuscript. All authors contributed to the article and approved the submitted version.

## Conflict of Interest

The authors declare that the research was conducted in the absence of any commercial or financial relationships that could be construed as a potential conflict of interest.

## Supplementary Material

Supplementary Figure 1 Phenotype transition of vascular smooth muscle cells (VSMCs) cocultured with extracellular vesicles (EVs) isolated from the primary cultured carotid arterial endothelial cells (ECs). **(A and D)** Representative Western blot gel documents showing the expression of PCNA and α-SMA induced by EVs collected from the carotid arterial ECs with (EVs-7-Ket) or without 7-Ket treatment (EVs-Ctrl). **(B,C,E,F)** showing the expression of PCNA and α-SMA induced by EVs collected from the carotid arterial ECs with (EVs-7-Ket) or without 7-Ket treatment (EVs-Ctrl).

Supplementary Figure 2 NLRP3 inflammasome formation and activation dose-dependently stimulated by 7-Ket) in the primary cultured carotid arterial ECs. **(A,B)** Representative fluorescent confocal microscope images displaying splite channel and the colocalization of NLRP3 (green) with ASC or caspase-1 (Red).

Supplementary Figure 3 NLRP3 inflammasome-dependent IL-1β secretion dose-dependently induced by 7-Ket *via* EVs in the primary cultured carotid arterial ECs. **(A,B)** Representative fluorescent confocal microscope images showing the colocalization of VPS16 (green) with IL-1β or Lamp-1 (Red).

Supplementary Figure 4 Effects of acid ceramidase (*AC*) and lysosome on the EVs release with IL-1β in the carotid arterial ECs. **(A)** Representative fluorescent confocal microscope images showing the colocalization of VPS16 (green) with IL-1β (Red).

Supplementary Figure 5 Effects of acid ceramidase (*AC*) and lysosome on the EVs release with IL-1β in the carotid arterial ECs. **(A)** Representative fluorescent confocal microscope images showing the colocalization of VPS16 (green) with Lamp-1 (Red).

Supplementary Figure 6 Effects of endothelial AC deficiency on the EVs secretion in the carotid arterial wall of mice. **(A)** Representative fluorescent confocal microscope images displaying the yellow dots or patches showing the colocalization of CD63 (green) with IL-1β (Red). **(B)** Representative fluorescent confocal microscope images showing the colocalization of VPS16 (green) with Lamp-1 (Red).

## References

[B1] Adhikari N. Shekar K. C. Staggs R. Win Z. Steucke K. Lin Y. W. (2015). Guidelines for the isolation and characterization of murine vascular smooth muscle cells. A report from the international society of cardiovascular translational research. *J. Cardiovasc. Transl. Res.* 8 158–163. 10.1007/s12265-015-9616-6 25788147 PMC5105830

[B2] Afewerki T. Ahmed S. Warren D. (2019). Emerging regulators of vascular smooth muscle cell migration. *J. Muscle Res. Cell Motil.* 40 185–196. 10.1007/s10974-019-09531-z 31254136 PMC6726670

[B3] Akyurek L. M. Yang Z. Y. Aoki K. San H. Nabel G. J. Parmacek M. S. (2000). SM22alpha promoter targets gene expression to vascular smooth muscle cells in vitro and in vivo. *Mol. Med.* 6 983–991. 10.1007/bf03401832 11147575 PMC1949920

[B4] Alexander M. R. Murgai M. Moehle C. W. Owens G. K. (2012). Interleukin-1beta modulates smooth muscle cell phenotype to a distinct inflammatory state relative to PDGF-DD via NF-kappaB-dependent mechanisms. *Physiol. Genom.* 44 417–429. 10.1152/physiolgenomics.00160.2011 22318995 PMC3339851

[B5] Arriola Benitez P. C. Pesce Viglietti A. I. Gomes M. T. R. Oliveira S. C. Quarleri J. F. Giambartolomei G. H. (2019). *Brucella abortus* infection elicited hepatic stellate cell-mediated fibrosis through inflammasome-dependent IL-1beta production. *Front. Immunol.* 10:3036 10.3389/fimmu.2019.03036 PMC6985094 32038610

[B6] Baixauli F. Lopez-Otin C. Mittelbrunn M. (2014). Exosomes and autophagy: coordinated mechanisms for the maintenance of cellular fitness. *Front. Immunol.* 5:403. 10.3389/fimmu.2014.00403 25191326 PMC4138502

[B7] Basatemur G. L. Jorgensen H. F. Clarke M. C. H. Bennett M. R. Mallat Z. (2019). Vascular smooth muscle cells in atherosclerosis. *Nat. Rev. Cardiol.* 16 727–744. 31243391 10.1038/s41569-019-0227-9

[B8] Beckmann N. Kadow S. Schumacher F. Gothert J. R. Kesper S. Draeger A. (2018). Pathological manifestations of Farber disease in a new mouse model. *Biol. Chem.* 399 1183–1202. 10.1515/hsz-2018-0170 29908121

[B9] Bennett M. R. Sinha S. Owens G. K. (2016). Vascular smooth muscle cells in atherosclerosis. *Circ. Res.* 118 692–702. 26892967 10.1161/CIRCRESAHA.115.306361 PMC4762053

[B10] Berezin A. E. Berezin A. A. (2020). Extracellular endothelial cell-derived vesicles: emerging role in cardiac and vascular remodeling in heart failure. *Front. Cardiovasc. Med.* 7:47 10.3389/fcvm.2020.00047 PMC7174683 32351973

[B11] Bergers G. Song S. (2005). The role of pericytes in blood-vessel formation and maintenance. *Neuro Oncol.* 7 452–464. 10.1215/s1152851705000232 16212810 PMC1871727

[B12] Bhat O. M. Li G. Yuan X. Huang D. Gulbins E. Kukreja R. C. (2020). Arterial medial calcification through enhanced small extracellular vesicle release in smooth muscle-specific asah1 gene knockout mice. *Sci. Rep.* 10:1645. 10.1038/s41598-020-58568-5 PMC6997457 32015399

[B13] Bianco F. Pravettoni E. Colombo A. Schenk U. Moller T. Matteoli M. (2005). Astrocyte-derived ATP induces vesicle shedding and IL-1 beta release from microglia. *J. Immunol.* 174 7268–7277. 10.4049/jimmunol.174.11.7268 15905573

[B14] Boulanger C. M. Loyer X. Rautou P. E. Amabile N. (2017). Extracellular vesicles in coronary artery disease. *Nat. Rev. Cardiol.* 14 259–272. 10.1038/nrcardio.2017.7 28150804

[B15] Buratta S. Tancini B. Sagini K. Delo F. Chiaradia E. Urbanelli L. (2020). Lysosomal exocytosis, exosome release and secretory autophagy: the autophagic- and endo-lysosomal systems go extracellular. *Int. J. Mol. Sci.* 21:2576. 10.3390/ijms21072576 32276321 PMC7178086

[B16] Calle P. Munoz A. Sola A. Hotter G. (2019). CPT1a gene expression reverses the inflammatory and anti-phagocytic effect of 7-ketocholesterol in RAW264.7 macrophages. *Lipids Health Dis.* 18:215. 10.1186/s12944-019-1156-7 PMC6902499 31823799

[B17] Chen X. Guo X. Ge Q. Zhao Y. Mu H. Zhang J. (2019). ER stress activates the NLRP3 inflammasome: a novel mechanism of atherosclerosis. *Oxid. Med. Cell Longev.* 2019:3462530. 10.1155/2019/3462530 PMC6800950 31687078

[B18] Chen Y. He X. Yuan X. Hong J. Bhat O. Li G. (2018). NLRP3 inflammasome formation and activation in nonalcoholic Steatohepatitis: therapeutic target for antimetabolic syndrome remedy FTZ. *Oxid. Med. Cell Longev.* 2018:2901871. 10.1155/2018/2901871 PMC6081604 30140364

[B19] Chen Y. Li X. Boini K. M. Pitzer A. L. Gulbins E. Zhang Y. (2015). Endothelial Nlrp3 inflammasome activation associated with lysosomal destabilization during coronary arteritis. *Biochim. Biophys. Acta* 1853 396–408. 10.1016/j.bbamcr.2014.11.012 25450976 PMC4289419

[B20] Chistiakov D. A. Orekhov A. N. Bobryshev Y. V. (2015a). Extracellular vesicles and atherosclerotic disease. *Cell Mol. Life. Sci.* 72 2697–2708. 25894694 10.1007/s00018-015-1906-2 PMC11113133

[B21] Chistiakov D. A. Orekhov A. N. Bobryshev Y. V. (2015b). Vascular smooth muscle cell in atherosclerosis. *Acta Physiol.* 214 33–50. 10.1111/apha.12466 25677529

[B22] Cypryk W. Nyman T. A. Matikainen S. (2018). From inflammasome to exosome-does extracellular vesicle secretion constitute an inflammasome-dependent immune response? *Front. Immunol.* 9:2188. 10.3389/fimmu.2018.02188 30319640 PMC6167409

[B23] Daniels M. J. Brough D. (2017). Unconventional pathways of secretion contribute to inflammation. *Int. J. Mol. Sci.* 18:102. 10.3390/ijms18010102 28067797 PMC5297736

[B24] Davies B. A. Lee J. R. Oestreich A. J. Katzmann D. J. (2009). Membrane protein targeting to the MVB/lysosome. *Chem. Rev.* 109 1575–1586. 10.1021/cr800473s 19243135 PMC3911787

[B25] Eitan E. Suire C. Zhang S. Mattson M. P. (2016). Impact of lysosome status on extracellular vesicle content and release. *Age. Res. Rev.* 32 65–74. 10.1016/j.arr.2016.05.001 27238186 PMC5154730

[B26] Eun S. Y. Ko Y. S. Park S. W. Chang K. C. Kim H. J. (2015). IL-1beta enhances vascular smooth muscle cell proliferation and migration via P2Y2 receptor-mediated RAGE expression and HMGB1 release. *Vascul. Pharmacol.* 72 108–117. 10.1016/j.vph.2015.04.013 25956731

[B27] Fader C. M. Sanchez D. Furlan M. Colombo M. I. (2008). Induction of autophagy promotes fusion of multivesicular bodies with autophagic vacuoles in k562 cells. *Traffic* 9 230–250. 10.1111/j.1600-0854.2007.00677.x 17999726

[B28] Gimbrone M. A. Jr. Garcia-Cardena G. (2016). Endothelial cell dysfunction and the pathobiology of atherosclerosis. *Circ. Res.* 118 620–636. 10.1161/circresaha.115.306301 26892962 PMC4762052

[B29] Hafiane A. Daskalopoulou S. S. (2018). Extracellular vesicles characteristics and emerging roles in atherosclerotic cardiovascular disease. *Metabolism* 85 213–222. 10.1016/j.metabol.2018.04.008 29727628

[B30] Hergenreider E. Heydt S. Treguer K. Boettger T. Horrevoets A. J. Zeiher A. M. (2012). Atheroprotective communication between endothelial cells and smooth muscle cells through miRNAs. *Nat. Cell Biol.* 14 249–256. 10.1038/ncb2441 22327366

[B31] Hessvik N. P. Llorente A. (2018). Current knowledge on exosome biogenesis and release. *Cell Mol. Life. Sci.* 75 193–208. 10.1007/s00018-017-2595-9 28733901 PMC5756260

[B32] Hong J. Bhat O. M. Li G. Dempsey S. K. Zhang Q. Ritter J. K. (2019). Lysosomal regulation of extracellular vesicle excretion during d-ribose-induced NLRP3 inflammasome activation in podocytes. *Biochim. Biophys. Acta Mol. Cell Res.* 1866 849–860. 10.1016/j.bbamcr.2019.02.007 30771382 PMC6800119

[B33] Huber L. A. Teis D. (2016). Lysosomal signaling in control of degradation pathways. *Curr. Opin. Cell Biol.* 39 8–14. 10.1016/j.ceb.2016.01.006 26827287

[B34] Jalil H. M. Ghazi H. F. (2020). NLRP3 Inflammasome gene polymorphisms variably associated with its serum levels in acute myocardial infarction. *Pak. J. Biol. Sci.* 23 612–618. 10.3923/pjbs.2020.612.618 32363817

[B35] Kapustin A. N. Chatrou M. L. Drozdov I. Zheng Y. Davidson S. M. Soong D. (2015). Vascular smooth muscle cell calcification is mediated by regulated exosome secretion. *Circ. Res.* 116 1312–1323. 10.1161/circresaha.116.305012 25711438

[B36] Karmakar M. Katsnelson M. A. Dubyak G. R. Pearlman E. (2016). Neutrophil P2X7 receptors mediate NLRP3 inflammasome-dependent IL-1beta secretion in response to ATP. *Nat. Commun.* 7:10555. 10.1038/ncomms10555 PMC4756306 26877061

[B37] Kim S. M. Huh J. W. Kim E. Y. Shin M. K. Park J. E. Kim S. W. (2019). Endothelial dysfunction induces atherosclerosis: increased aggrecan expression promotes apoptosis in vascular smooth muscle cells. *BMB Rep.* 52 145–150. 10.5483/bmbrep.2019.52.2.282 30638179 PMC6443320

[B38] Kingsbury S. R. Conaghan P. G. Mcdermott M. F. (2011). The role of the NLRP3 inflammasome in gout. *J. Inflamm. Res.* 4 39–49. 10.2147/jir.s11330 22096368 PMC3218743

[B39] Kobayashi M. Inoue K. Warabi E. Minami T. Kodama T. (2005). A simple method of isolating mouse aortic endothelial cells. *J. Atheroscler. Thromb.* 12 138–142. 10.5551/jat.12.138 16020913

[B40] Koka S. Xia M. Chen Y. Bhat O. M. Yuan X. Boini K. M. (2017). Endothelial NLRP3 inflammasome activation and arterial neointima formation associated with acid sphingomyelinase during hypercholesterolemia. *Redox Biol.* 13 336–344. 10.1016/j.redox.2017.06.004 28633109 PMC5479959

[B41] Korn C. Scholz B. Hu J. Srivastava K. Wojtarowicz J. Arnsperger T. (2014). Endothelial cell-derived non-canonical Wnt ligands control vascular pruning in angiogenesis. *Development* 141 1757–1766. 10.1242/dev.104422 24715464

[B42] Laurier-Laurin M. E. De Montigny A. Attiori Essis S. Cyr M. Massicotte G. (2014). Blockade of lysosomal acid ceramidase induces GluN2B-dependent Tau phosphorylation in rat hippocampal slices. *Neural Plast.* 2014: pgrng196812. 10.1155/2014/196812 PMC4170924 25276436

[B43] Li G. Chen Z. Bhat O. M. Zhang Q. Abais-Battad J. M. Conley S. M. (2017). NLRP3 inflammasome as a novel target for docosahexaenoic acid metabolites to abrogate glomerular injury. *J. Lipid Res.* 58 1080–1090. 10.1194/jlr.m072587 28404641 PMC5454504

[B44] Li G. Huang D. Hong J. Bhat O. M. Yuan X. Li P. L. (2019). Control of lysosomal TRPML1 channel activity and exosome release by acid ceramidase in mouse podocytes. *Am. J. Physiol. Cell Physiol.* 317 C481–C491. 31268777 10.1152/ajpcell.00150.2019 PMC6766620

[B45] Li M. Qian M. Kyler K. Xu J. (2018). Endothelial-vascular smooth muscle cells interactions in atherosclerosis. *Front. Cardiovasc. Med.* 5:151. 10.3389/fcvm.2018.00151 30406116 PMC6207093

[B46] Li X. Han W. Q. Boini K. M. Xia M. Zhang Y. Li P. L. (2013). TRAIL death receptor 4 signaling via lysosome fusion and membrane raft clustering in coronary arterial endothelial cells: evidence from ASM knockout mice. *J. Mol. Med.* 91 25–36. 10.1007/s00109-012-0968-y 23108456 PMC3537912

[B47] Li X. Zhang Y. Xia M. Gulbins E. Boini K. M. Li P. L. (2014). Activation of Nlrp3 inflammasomes enhances macrophage lipid-deposition and migration: implication of a novel role of inflammasome in atherogenesis. *PLoS One* 9:e87552. 10.1371/journal.pone.0087552 24475307 PMC3903678

[B48] Liang C. C. Park A. Y. Guan J. L. (2007). In vitro scratch assay: a convenient and inexpensive method for analysis of cell migration in vitro. *Nat. Protoc.* 2 329–333. 10.1038/nprot.2007.30 17406593

[B49] Liebner S. Cavallaro U. Dejana E. (2006). The multiple languages of endothelial cell-to-cell communication. *Arterioscler. Thromb. Vasc. Biol.* 26 1431–1438. 10.1161/01.atv.0000218510.04541.5e 16556854

[B50] Lin X. Li S. Wang Y. J. Wang Y. Zhong J. Y. He J. Y. (2019). Exosomal Notch3 from high glucose-stimulated endothelial cells regulates vascular smooth muscle cells calcification/aging. *Life Sci.* 232:116582. 10.1016/j.lfs.2019.116582 31220525

[B51] Liu R. Shen H. Ma J. Sun L. Wei M. (2016). Extracellular vesicles derived from adipose mesenchymal stem cells regulate the phenotype of smooth muscle cells to limit intimal hyperplasia. *Cardiovasc. Drugs. Ther.* 30 111–118. 10.1007/s10557-015-6630-5 26650931

[B52] Lopez-Castejon G. Brough D. (2011). Understanding the mechanism of IL-1beta secretion. *Cytokine Growth Factor. Rev.* 22 189–195. 10.1016/j.cytogfr.2011.10.001 22019906 PMC3714593

[B53] Lu B. Zhong J. Pan J. Yuan X. Ren M. Jiang L. (2019). Gdf11 gene transfer prevents high fat diet-induced obesity and improves metabolic homeostasis in obese and STZ-induced diabetic mice. *J. Transl. Med.* 17:422. 10.1186/s12967-019-02166-1 PMC6915940 31847906

[B54] Lutter S. Xie S. Tatin F. Makinen T. (2012). Smooth muscle-endothelial cell communication activates Reelin signaling and regulates lymphatic vessel formation. *J. Cell Biol.* 197 837–849. 10.1083/jcb.201110132 22665518 PMC3373399

[B55] Lyle A. N. Taylor W. R. (2019). The pathophysiological basis of vascular disease. *Lab. Invest.* 99 284–289. 10.1038/s41374-019-0192-2 30755702

[B56] MacKenzie A. Wilson H. L. Kiss-Toth E. Dower S. K. North R. A. Surprenant A. (2001). Rapid secretion of interleukin-1beta by microvesicle shedding. *Immunity* 15 825–835. 10.1016/s1074-7613(01)00229-1 11728343

[B57] Martin-Sanchez F. Diamond C. Zeitler M. Gomez A. I. Baroja-Mazo A. Bagnall J. (2016). Inflammasome-dependent IL-1beta release depends upon membrane permeabilisation. *Cell Death Differ.* 23 1219–1231. 10.1038/cdd.2015.176 26868913 PMC4946890

[B58] Mo D. Zeng G. Yuan X. Chen M. Hu L. Li H. (2018). Molecular docking simulation on the interactions of laccase from *Trametes versicolor* with nonylphenol and octylphenol isomers. *Bioprocess Biosyst. Eng.* 41 331–343. 10.1007/s00449-017-1866-z 29185034

[B59] Niu C. Wang X. Zhao M. Cai T. Liu P. Li J. (2016). Macrophage foam cell-derived extracellular vesicles promote vascular smooth muscle cell migration and adhesion. *J. Am. Heart Assoc.* 5:e004099. 10.1161/JAHA.116.004099 PMC5121506 27792649

[B60] Noble J. M. Roberts L. M. Vidavsky N. Chiou A. E. Fischbach C. Paszek M. J. (2020). Direct comparison of optical and electron microscopy methods for structural characterization of extracellular vesicles. *J. Struct. Biol.* 210:107474. 10.1016/j.jsb.2020.107474 32032755 PMC7067680

[B61] Otani K. Yokoya M. Fujioka Y. Okada M. Yamawaki H. (2020). Small extracellular vesicles from rat plasma promote migration and proliferation of vascular smooth muscle cells. *J. Vet. Med. Sci.* 82 299–306. 10.1292/jvms.19-0643 31902833 PMC7118471

[B62] Park J. H. Schuchman E. H. (2006). Acid ceramidase and human disease. *Biochim. Biophys. Acta* 1758 2133–2138. 17064658 10.1016/j.bbamem.2006.08.019

[B63] Peiro C. Redondo J. Rodriguez-Martinez M. A. Angulo J. Marin J. Sanchez-Ferrer C. F. (1995). Influence of endothelium on cultured vascular smooth muscle cell proliferation. *Hypertension* 25 748–751. 10.1161/01.hyp.25.4.748 7721427

[B64] Piccoli E. Nadai M. Caretta C. M. Bergonzini V. Del Vecchio C. Ha H. R. (2011). Amiodarone impairs trafficking through late endosomes inducing a Niemann-Pick C-like phenotype. *Biochem. Pharmacol.* 82 1234–1249. 10.1016/j.bcp.2011.07.090 21878321 PMC7092840

[B65] Pitulescu M. E. Adams R. H. (2014). Regulation of signaling interactions and receptor endocytosis in growing blood vessels. *Cell Adh. Migr.* 8 366–377. 10.4161/19336918.2014.970010 25482636 PMC4594521

[B66] Pizzirani C. Ferrari D. Chiozzi P. Adinolfi E. Sandona D. Savaglio E. (2007). Stimulation of P2 receptors causes release of IL-1beta-loaded microvesicles from human dendritic cells. *Blood* 109 3856–3864. 10.1182/blood-2005-06-031377 17192399

[B67] Price P. A. Buckley J. R. Williamson M. K. (2001). The amino bisphosphonate ibandronate prevents vitamin D toxicity and inhibits vitamin D-induced calcification of arteries, cartilage, lungs and kidneys in rats. *J. Nutr.* 131 2910–2915. 10.1093/jn/131.11.2910 11694617

[B68] Qi Y. X. Jiang J. Jiang X. H. Wang X. D. Ji S. Y. Han Y. (2011). PDGF-BB and TGF-{beta}1 on cross-talk between endothelial and smooth muscle cells in vascular remodeling induced by low shear stress. *Proc. Natl. Acad. Sci. U.S.A.* 108 1908–1913. 10.1073/pnas.1019219108 21245329 PMC3033274

[B69] Qu Y. Franchi L. Nunez G. Dubyak G. R. (2007). Nonclassical IL-1 beta secretion stimulated by P2X7 receptors is dependent on inflammasome activation and correlated with exosome release in murine macrophages. *J. Immunol.* 179 1913–1925. 10.4049/jimmunol.179.3.1913 17641058

[B70] Ren X. S. Tong Y. Qiu Y. Ye C. Wu N. Xiong X. Q. (2020). MiR155-5p in adventitial fibroblasts-derived extracellular vesicles inhibits vascular smooth muscle cell proliferation via suppressing angiotensin-converting enzyme expression. *J. Extracell Ves.* 9:1698795. 10.1080/20013078.2019.1698795 31839907 PMC6896498

[B71] Ryu J. H. Jeon E. Y. Kim S. J. (2019). Indoxyl sulfate-induced extracellular vesicles released from endothelial cells stimulate vascular smooth muscle cell proliferation by inducing transforming growth factor-beta production. *J. Vasc. Res.* 56 129–138. 10.1159/000496796 31085925

[B72] Serban K. A. Rezania S. Petrusca D. N. Poirier C. Cao D. Justice M. J. (2016). Structural and functional characterization of endothelial microparticles released by cigarette smoke. *Sci. Rep.* 6:31596. 10.1038/srep31596 PMC4987682 27530098

[B73] Sharma H. Chinnappan M. Agarwal S. Dalvi P. Gunewardena S. O’brien-Ladner A. (2018). Macrophage-derived extracellular vesicles mediate smooth muscle hyperplasia: role of altered miRNA cargo in response to HIV infection and substance abuse. *FASEB J.* 32 5174–5185. 10.1096/fj.201701558r 29672222 PMC6103174

[B74] Shi G. Chen S. Wandu W. S. Ogbeifun O. Nugent L. F. Maminishkis A. (2015). Inflammasomes induced by 7-Ketocholesterol and other stimuli in RPE and in bone marrow-derived cells differ markedly in their production of IL-1beta and IL-18. *Invest. Ophthalmol. Vis. Sci.* 56 1658–1664. 10.1167/iovs.14-14557 25678688 PMC4354246

[B75] Sokolova M. Sjaastad I. Louwe M. C. Alfsnes K. Aronsen J. M. Zhang L. (2019). NLRP3 inflammasome promotes myocardial remodeling during diet-induced obesity. *Front. Immunol.* 10:1621. 10.3389/fimmu.2019.01621 31379826 PMC6648799

[B76] Swatler J. Dudka W. Piwocka K. (2020). Isolation and characterization of extracellular vesicles from cell culture conditioned medium for immunological studies. *Curr. Protoc. Immunol.* 129:e96. 10.1002/cpim.96 32453501

[B77] Tahir H. Niculescu I. Bona-Casas C. Merks R. M. Hoekstra A. G. (2015). An in silico study on the role of smooth muscle cell migration in neointimal formation after coronary stenting. *J. R. Soc. Interf.* 12:20150358. 10.1098/rsif.2015.0358 26063828 PMC4528603

[B78] Vacante F. Denby L. Sluimer J. C. Baker A. H. (2019). The function of miR-143, miR-145 and the MiR-143 host gene in cardiovascular development and disease. *Vascul. Pharmacol.* 112 24–30. 10.1016/j.vph.2018.11.006 30502421 PMC6395947

[B79] Wagenseil J. E. Mecham R. P. (2009). Vascular extracellular matrix and arterial mechanics. *Physiol. Rev.* 89 957–989. 10.1152/physrev.00041.2008 19584318 PMC2775470

[B80] Wan Z. Fan Y. Liu X. Xue J. Han Z. Zhu C. (2019). NLRP3 inflammasome promotes diabetes-induced endothelial inflammation and atherosclerosis. *Diabetes Metab. Syndr. Obes.* 12 1931–1942. 10.2147/dmso.s222053 31571967 PMC6759984

[B81] Wang L. Chen Y. Li X. Zhang Y. Gulbins E. Zhang Y. (2016). Enhancement of endothelial permeability by free fatty acid through lysosomal cathepsin B-mediated Nlrp3 inflammasome activation. *Oncotarget* 7 73229–73241. 10.18632/oncotarget.12302 27689324 PMC5341975

[B82] Wang S. Zhan J. Lin X. Wang Y. Wang Y. Liu Y. (2020). CircRNA-0077930 from hyperglycaemia-stimulated vascular endothelial cell exosomes regulates senescence in vascular smooth muscle cells. *Cell Biochem. Funct.* 38, 1056–1068. 10.1002/cbf.3543 32307741

[B83] Wartosch L. Gunesdogan U. Graham S. C. Luzio J. P. (2015). Recruitment of VPS33A to HOPS by VPS16 is required for lysosome fusion with Endosomes and Autophagosomes. *Traffic* 16 727–742. 10.1111/tra.12283 25783203 PMC4510706

[B84] Xia M. Boini K. M. Abais J. M. Xu M. Zhang Y. Li P. L. (2014). Endothelial NLRP3 inflammasome activation and enhanced neointima formation in mice by adipokine visfatin. *Am. J. Pathol.* 184 1617–1628. 10.1016/j.ajpath.2014.01.032 24631027 PMC4005976

[B85] Xing J. H. Li R. Gao Y. Q. Wang M. Y. Liu Y. Z. Hong J. (2019). NLRP3 inflammasome mediate palmitate-induced endothelial dysfunction. *Life Sci.* 239:116882. 10.1016/j.lfs.2019.116882 31705915

[B86] Xing Z. Zhao C. Liu H. Fan Y. (2020). Endothelial progenitor cell-derived extracellular vesicles: a novel candidate for regenerative medicine and disease treatment. *Adv. Healthc. Mater.* 9:e2000255. 10.1002/adhm.202000255 32378361

[B87] Yu J. Zhang Y. Zhang X. Rudic R. D. Bauer P. M. Altieri D. C. (2012). Endothelium derived nitric oxide synthase negatively regulates the PDGF-survivin pathway during flow-dependent vascular remodeling. *PLoS One* 7:e31495. 10.1371/journal.pone.0031495 22355372 PMC3280303

[B88] Yuan X. Bhat O. M. Lohner H. Li N. Zhang Y. Li P. L. (2019a). Inhibitory effects of growth differentiation factor 11 on autophagy deficiency-induced dedifferentiation of arterial smooth muscle cells. *Am. J. Physiol. Heart Circ. Physiol.* 316 H345–H356. 30462553 10.1152/ajpheart.00342.2018 PMC6397385

[B89] Yuan X. Bhat O. M. Lohner H. Zhang Y. Li P. L. (2019b). Endothelial acid ceramidase in exosome-mediated release of NLRP3 inflammasome products during hyperglycemia: evidence from endothelium-specific deletion of Asah1 gene. *Biochim. Biophys. Acta Mol. Cell Biol. Lipids* 1864:158532. 10.1016/j.bbalip.2019.158532 31647995 PMC6909250

[B90] Yuan X. Bhat O. M. Meng N. Lohner H. Li P. L. (2018a). Protective role of autophagy in Nlrp3 inflammasome activation and medial thickening of mouse coronary arteries. *Am. J. Pathol.* 188 2948–2959. 10.1016/j.ajpath.2018.08.014 30273598 PMC6334256

[B91] Yuan X. Wang L. Bhat O. M. Lohner H. Li P. L. (2018b). Differential effects of short chain fatty acids on endothelial Nlrp3 inflammasome activation and neointima formation: antioxidant action of butyrate. *Redox Biol.* 16 21–31. 10.1016/j.redox.2018.02.007 29475132 PMC5842312

[B92] Zhang Y. Chen Y. Li P. L. Li X. (2019). Contribution of cathepsin B-dependent Nlrp3 inflammasome activation to nicotine-induced endothelial barrier dysfunction. *Eur. J. Pharmacol.* 865:172795. 10.1016/j.ejphar.2019.172795 31733211 PMC6925381

[B93] Zhang Y. Li X. Pitzer A. L. Chen Y. Wang L. Li P. L. (2015). Coronary endothelial dysfunction induced by nucleotide oligomerization domain-like receptor protein with pyrin domain containing 3 inflammasome activation during hypercholesterolemia: beyond inflammation. *Antioxid. Redox Signal.* 22 1084–1096. 10.1089/ars.2014.5978 25739025 PMC4403230

[B94] Zhang Y. Xu M. Xia M. Li X. Boini K. M. Wang M. (2014). Defective autophagosome trafficking contributes to impaired autophagic flux in coronary arterial myocytes lacking CD38 gene. *Cardiovasc. Res.* 102 68–78. 10.1093/cvr/cvu011 24445604 PMC3958620

[B95] Zhao L. Luo H. Li X. Li T. He J. Qi Q. (2017). Exosomes derived from human pulmonary artery endothelial cells shift the balance between proliferation and *Apoptosis* of smooth muscle cells. *Cardiology* 137 43–53. 10.1159/000453544 28068653

[B96] Zheng B. Yin W. N. Suzuki T. Zhang X. H. Zhang Y. Song L. L. (2017). Exosome-mediated miR-155 transfer from smooth muscle cells to endothelial cells induces endothelial injury and promotes atherosclerosis. *Mol. Ther.* 25 1279–1294. 10.1016/j.ymthe.2017.03.031 28408180 PMC5475247

[B97] Zhuang T. Liu J. Chen X. Zhang L. Pi J. Sun H. (2019). Endothelial Foxp1 suppresses atherosclerosis via modulation of Nlrp3 inflammasome activation. *Circ. Res.* 125 590–605. 10.1161/circresaha.118.314402 31318658

