# Supplementary figures and images for: Reversal of Endothelial Extracellular Vesicle-Induced Smooth Muscle Phenotype Transition by Hypercholesterolemia Stimulation: Role of NLRP3 Inflammasome Activation
Source: Front Cell Dev Biol. 2020 Dec 21;8:597423. doi: 10.3389/fcell.2020.597423 (PMC7779768; doi:10.3389/fcell.2020.597423)

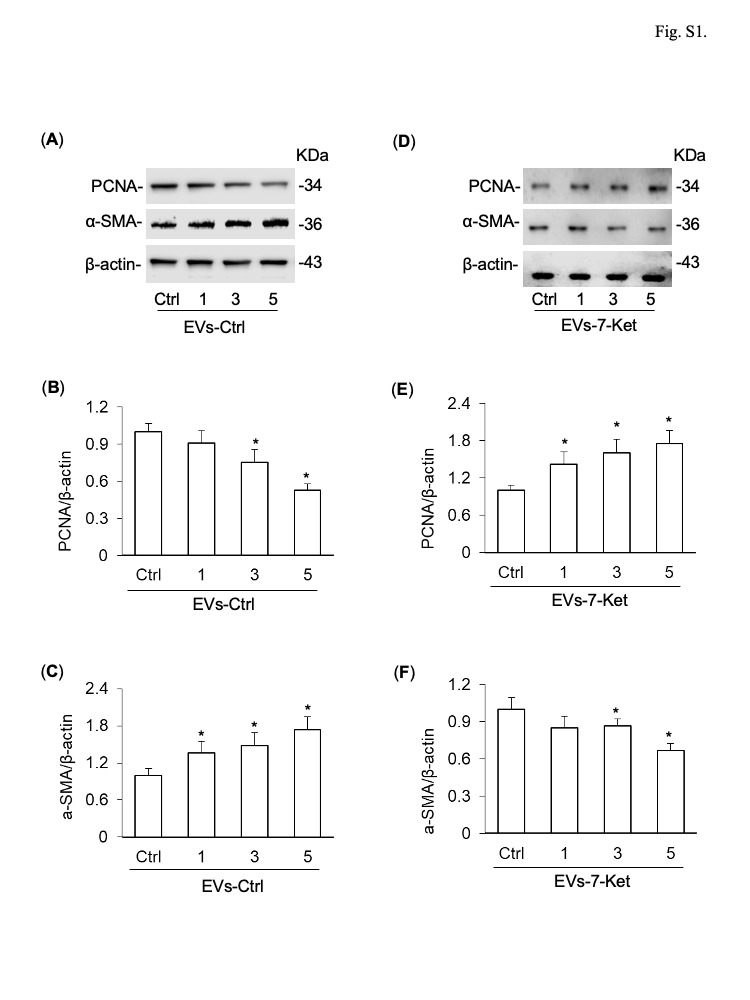

Supplement: Supplementary Figure 1 — Phenotype transition of vascular smooth muscle cells (VSMCs) cocultured with extracellular vesicles (EVs) isolated from the primary cultured carotid arterial endothelial cells (ECs). (A and D) Representative Western blot gel documents showing the expression of PCNA and α-SMA induced by EVs collected from the carotid arterial ECs with (EVs-7-Ket) or without 7-Ket treatment (EVs-Ctrl). (B,C,E,F) showing the expression of PCNA and α-SMA induced by EVs collected from the carotid arterial ECs with (EVs-7-Ket) or without 7-Ket treatment (EVs-Ctrl). [file Image_1.tiff]

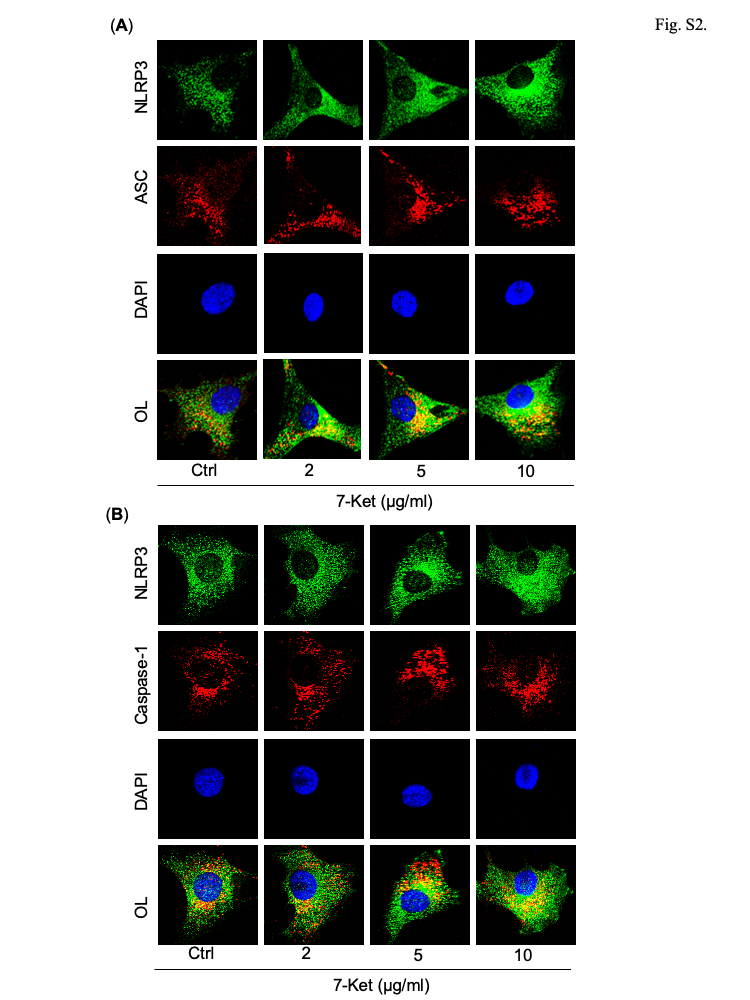

Supplement: Supplementary Figure 2 — NLRP3 inflammasome formation and activation dose-dependently stimulated by 7-Ket) in the primary cultured carotid arterial ECs. (A,B) Representative fluorescent confocal microscope images displaying splite channel and the colocalization of NLRP3 (green) with ASC or caspase-1 (Red). [file Image_2.tiff]

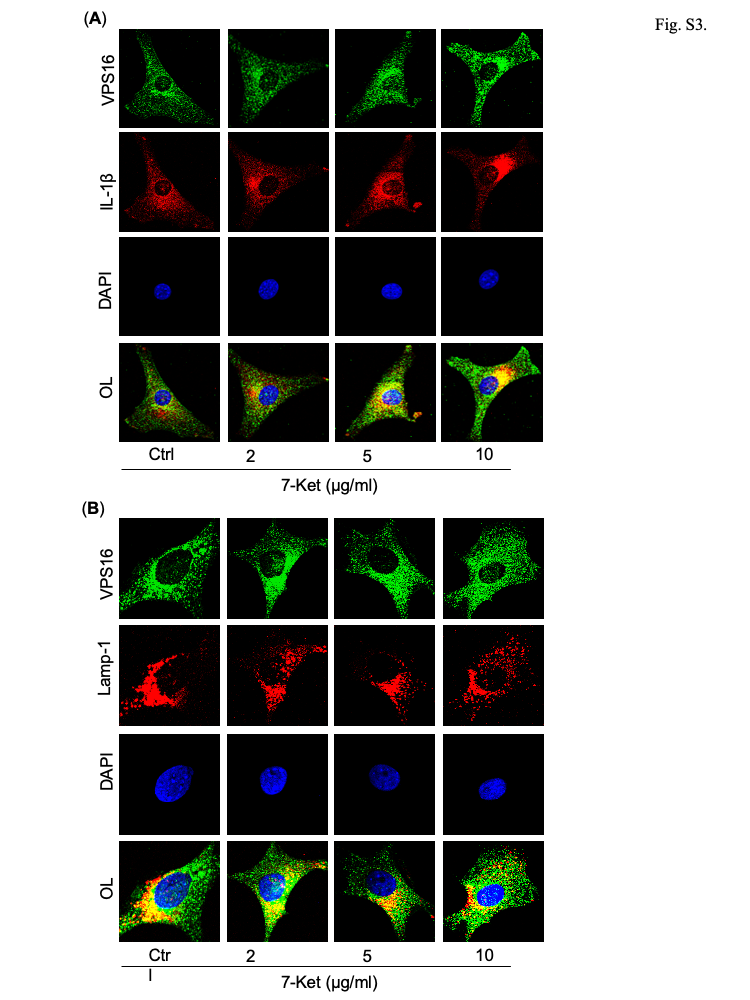

Supplement: Supplementary Figure 3 — NLRP3 inflammasome-dependent IL-1β secretion dose-dependently induced by 7-Ket via EVs in the primary cultured carotid arterial ECs. (A,B) Representative fluorescent confocal microscope images showing the colocalization of VPS16 (green) with IL-1β or Lamp-1 (Red). [file Image_3.tiff]

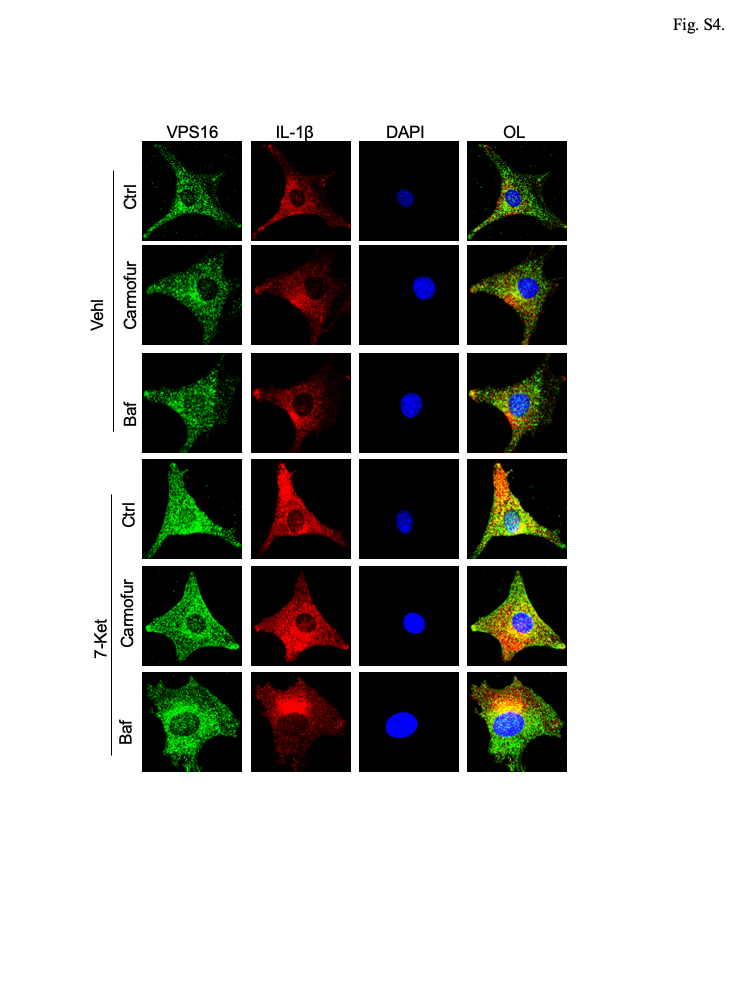

Supplement: Supplementary Figure 4 — Effects of acid ceramidase (AC) and lysosome on the EVs release with IL-1β in the carotid arterial ECs. (A) Representative fluorescent confocal microscope images showing the colocalization of VPS16 (green) with IL-1β (Red). [file Image_4.tiff]

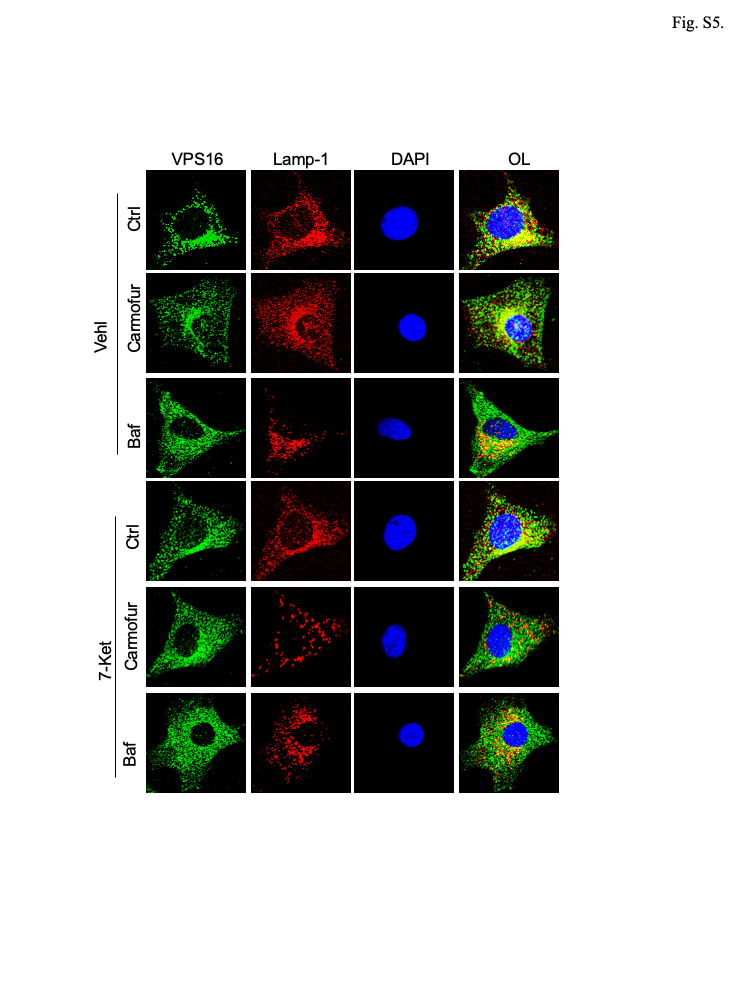

Supplement: Supplementary Figure 5 — Effects of acid ceramidase (AC) and lysosome on the EVs release with IL-1β in the carotid arterial ECs. (A) Representative fluorescent confocal microscope images showing the colocalization of VPS16 (green) with Lamp-1 (Red). [file Image_5.tiff]

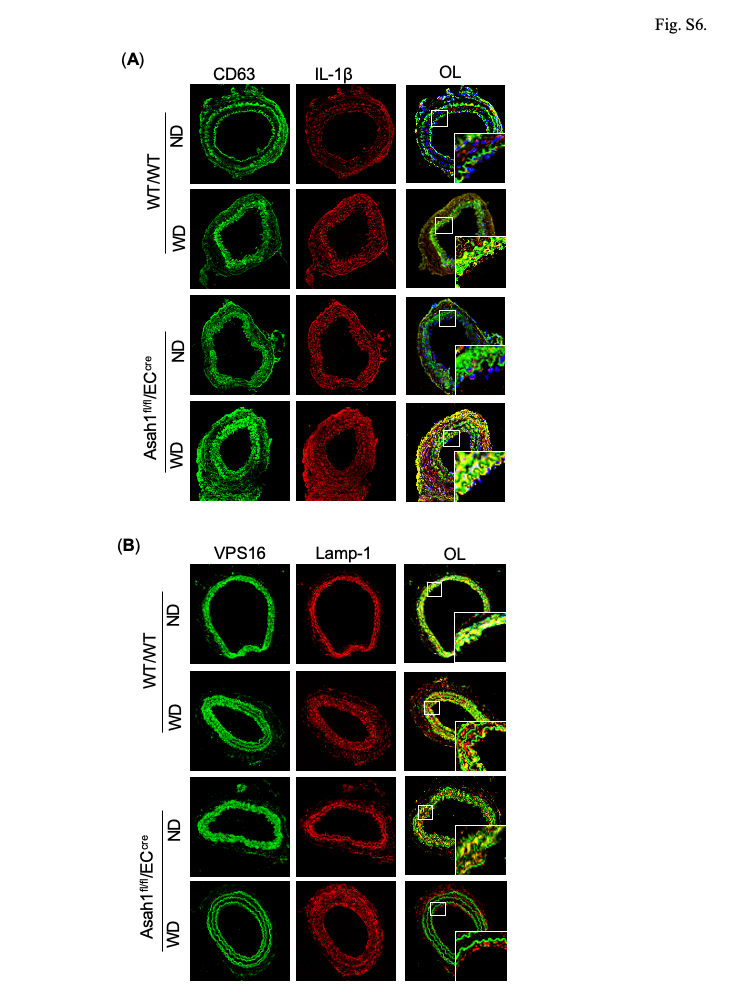

Supplement: Supplementary Figure 6 — Effects of endothelial AC deficiency on the EVs secretion in the carotid arterial wall of mice. (A) Representative fluorescent confocal microscope images displaying the yellow dots or patches showing the colocalization of CD63 (green) with IL-1β (Red). (B) Representative fluorescent confocal microscope images showing the colocalization of VPS16 (green) with Lamp-1 (Red). [file Image_6.tiff]
